# Anomeric sugar boronic acid analogues as potential agents for boron neutron capture therapy

**DOI:** 10.3762/bjoc.15.135

**Published:** 2019-06-19

**Authors:** Daniela Imperio, Erika Del Grosso, Silvia Fallarini, Grazia Lombardi, Luigi Panza

**Affiliations:** 1 Department of Pharmaceutical Sciences, Università del Piemonte Orientale, Largo Donegani 2, 28100 Novara, Italy

**Keywords:** antitumor agents, boron neutron capture therapy, boronic acid, hydroboration, sugar analogue

## Abstract

After the development of accelerators as neutron source, the access to new suitable agents for boron neutron capture therapy (BNCT) became a major need. Among many others, sugar boronic acids have recently attracted attention as boron carriers. Herein we report the synthesis and preliminary biological studies of two new sugar analogues containing a boronic acid at the anomeric position. The analogues were obtained by hydroboration of proper open-chain terminal alkenes that, after quenching with water, spontaneously afforded cyclic boronic acids with hemiacetal-like structures.

## Introduction

Boron neutron capture therapy (BNCT) belongs to the so-called binary therapies for cancer treatment. It is based on the fission reaction after a low-energy neutron capture by a ^10^B atom. The neutron capture reaction gives rise to two high linear energy transfer (LET) particles (an α-particle and a lithium ion) that dissipate all their energy in a path of the order of a cell diameter (5–9 μm) in biological tissues, which gives them the potential for precise cell damage. Highly selective delivery, accumulation of boron in tumor tissues and proper subcellular distribution are required to achieve a successful neutron capture therapy [1].

^10^B atoms to be administered should be incorporated into non-toxic structures. So far, the two compounds currently used for clinical trials are sodium mercaptoundecahydrododecaborate (Na_2_B_12_H_11_SH, BSH) and ʟ-boronophenylalanine (BPA). However, these two compounds are not fully satisfactory owing to their rapid clearance from blood [2], their only moderate selectivity for cancer cells and short retention times in tumors. Therefore, research efforts are ongoing to synthesize new BNCT candidates with improved tumor selectivity and cell retention.

The development of boron-containing carbohydrate derivatives for BNCT has received special attention because of the sugar-preferential uptake by tumor cells [3]. Tumor cells perform glycolysis at a faster rate than their noncancerous tissue counterparts, so increasing the glucose consumption with respect to healthy cells. Galactose and fructose also allow tumor growth in the absence of glucose.

Boronic acid derivatives have gained interest in the last years in different fields such as the development of enzyme inhibitors, drug delivery polymers, saccharide sensors and as boron carriers for BNCT, e.g., amino acid derivatives or DNA ligands [4–5].

In the frame of a more general research program, we have recently published the synthesis of boronic acids derivatives of monosaccharides in which one of the sugar hydroxy groups is substituted by a boronic acid moiety [6]. A related approach has been described [7] recently, where, among other derivatives, 1,2-dideoxy-ᴅ-glucopyranos-2-ylboronic acid has been synthesized through a regio- and stereoselective hydroboration of persilylated glucal.

**Figure 1:**
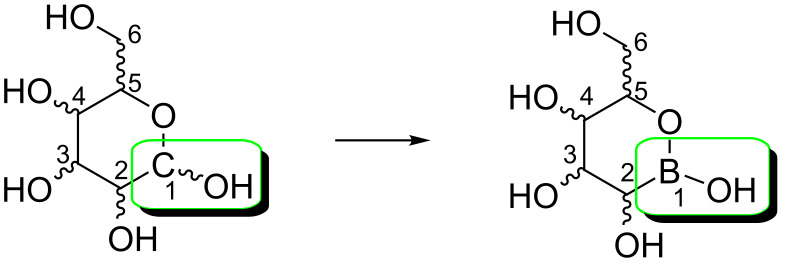
Structure of boronic acid analogues (for clarity, sugar numbering has been conserved into the analogues).

Considering the different substitution positions for the boronic acid in the sugar skeleton, we were curious to observe the chemical and biological behavior of novel derivatives containing this functional group at the anomeric position, giving rise to an isostere analogue able to mimic the monosaccharide (see Figure 1).

## Results and Discussion

In the present paper the synthesis of two analogues bearing a boron atom at the anomeric position is reported. These model compounds, never described before in literature, allowed to perform a preliminary biological evaluation as well as an evaluation of their stability.

The compatibility of boronic acids/esters in the presence of other functional groups has been reviewed [8]. Usually, β-alkoxyboronates are reasonably stable with some exceptions [9], while β-hydroxyboronic acids are prone to elimination. However, β-hydroxyboronic acids are described, such as in the case of the previously mentioned glucopyranos-2-ylboronic acid [7] as well as in other reports where they are usually isolated after conversion into trifluoroborates [10].

In principle, the best way to obtain anomeric boron analogues mimicking hexoses would be the addition of a nucleophilic boron reagent on a properly protected pentose. Actually, boryllithium and magnesium reagents [11–14] have been described but, after a careful evaluation, we discarded this approach considering the scarce information on the general applicability of these reagents and for the manipulations required after the addition to get the desired boronic acid.

**Figure 2:**
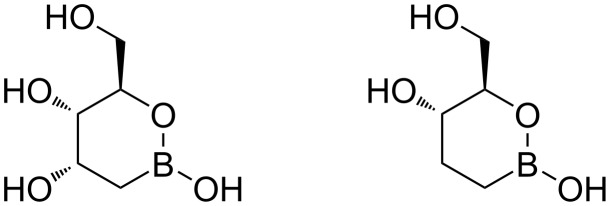
Structures of boron analogues.

Therefore, we decided to explore the synthesis of two simplified compounds, namely the 2-deoxy and the 2,3-dideoxy derivatives, whose structures are reported in Figure 2, in order to have a reliable synthesis and to get preliminary information on their stability.

**Figure 3:**
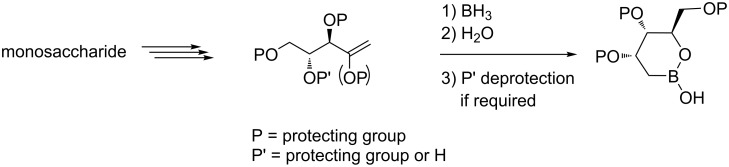
Synthetic strategy.

We planned a strategy based on a hydroboration reaction of terminal, suitably functionalized alkenes, which can be obtained from properly protected sugar precursors (Figure 3).

**Scheme 1:**
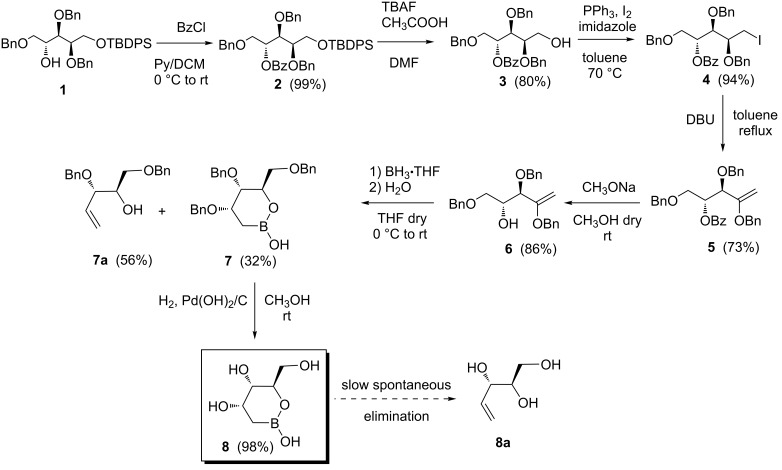
Synthesis of 2-deoxy analogue **8**.

The synthesis of the first analogue **8**, shown in Scheme 1, started from known compound **1**, that is easily obtained from 2,3,5-tri-*O*-benzyl-ᴅ-arabinose according to a literature procedure [15]. Benzoylation of the free hydroxy group of **1**, followed by removal of the silyl protecting group gave the intermediate **3** in good yield. Standard substitution of the hydroxy group gave the 1-deoxy-1-iododerivative **4**. Base-promoted dehydroiodination, followed by Zémplen removal of the benzoate gave the desired enol ether **6**, which was selected as precursor for the preparation of the first anomeric boron analogue.

Different conditions and reagents were tested for the hydroboration of the enol ethers **5** and **6** (including the use of catecholborane or dibromoborane at low temperature), but complex mixtures containing elimination byproducts were obtained in any condition. We were able to obtain the desired product **7** by the simple treatment of **6** with an excess of borane-THF complex (5 molar equiv) followed by quenching with water, while in the same conditions compound **5** again gave extensive decomposition. These conditions allowed the isolation of the cyclic anomeric boron analogue **7** in moderate yield. Unfortunately, we isolated as main side product the known elimination compound **7a** [16], which further confirms the strong tendency of β-alkoxyboronic acids to give rise to elimination.

The stereochemistry of the newly formed stereocenter was inferred from the coupling constant between H-3 and H-4 (sugar numbering). In fact, the measured *J*_3,4_ value was 1.8 Hz, which is only compatible with a *ribo* configuration of the compound **7** [17]. Moreover, the coupling constants of 4.6 and 5.7 Hz of H-3 with the protons at C-2 further confirm the assigned C-3 configuration. We were unable to isolate the *arabino*-configurated epimer from the reaction mixture, which, if present, may therefore be formed in a very small amount. As expected, no regioisomer was detected, due to the contribution of both steric and electronic effects on the hydroboration at C-1 [7].

**Figure 4:**
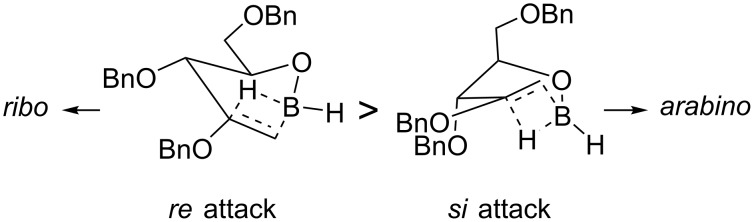
Postulated transition states for the hydroboration reaction.

In order to explain the stereoselectivity in the hydroboration reaction we hypothesized the transition states bearing to the two epimers. We assumed that borane would react firstly with the free hydroxy group generating an intermediate alkoxyborane, and that the hydroboration reaction occurs intramolecularly on such intermediate. Based on these premises, two transition states can be identified (Figure 4) for the hydroboration reaction on each of the two diastereotopic faces of the double bond. The transition state deriving from the attack on the *si* face, which leads to the *arabino*-configurated product, contains two destabilizing pseudoaxial substituents. The alternative, a more stable transition state formed from the *re*-face attack would bear to the actually obtained 3*S* configurated boronic acid analogue **7**.

The final deprotection of compound **7** by conventional catalytic hydrogenolysis gave the final compound **8** in almost quantitative yield. Again, the final product proved to be unstable towards elimination giving rise slowly to the corresponding alkene **8a** [18]. In fact, when compound **8** was kept in water or methanol solution at room temperature, the formation of **8a** was observed in about 20% within a few hours. Moreover, the same elimination occurs even when **8** was stored at −20 °C.

**Scheme 2:**
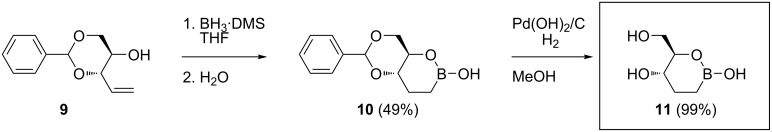
Synthesis of 2,3-dideoxy analogue **11**.

To avoid β-elimination we planned the synthesis of a new final sugar devoid of the hydroxy group at position 3. Therefore, we selected the alkene **9** as substrate for the hydroboration reaction (Scheme 2).

The hydroboration reaction of the known compound **9** [19], again with 5 equiv of reagent, afforded the cyclic boronic acid **10** in a fair yield. Benzylidene group hydrogenolysis gave the deprotected pure cyclic boronic acid **11** in quantitative yield and compound **11** proved to be stable for months. In this case the elimination reaction was not observed as expected for a 3-deoxy derivative. Moreover, compound **11** also proved to be stable towards oxidation of the C–B bond which could occur on boronic acids.

Inspection of the NMR spectra can give some information on the conformation of cyclic boronic acids. Considering that the boron atom should be sp^2^ hybridized it is not possible to assign any α/β configuration to the position 1 of the sugar analogue. Compound **7** showed the following coupling constants: *J*_2a,3_ = 4.6 Hz, *J*_2b,3_ = 5.7 Hz, *J*_3,4_ = 1.8 Hz and *J*_4,5_ = 7.2 Hz. From these values, a chair-like conformation could be inferred, with some flattening around the boron atom. Compound **8** shows two isochronal protons at C-2 with a *J*_2,3_ = 4.9 Hz, and superposition of protons H-3 and H-5 not allowing to hypothesize a clear picture of the 6-membered ring conformation. Concerning compound **10**, the molecule is rigid around the benzylidene ring, while a broadening of the H-2, H-3 and H-4 signals suggest a slow equilibrium between different conformations. After deprotection of the benzylidene group in addition to line broadening, signal superposition is observed, hampering any conclusion on the conformation of compound **11**.

In order to get more information about the solution behavior of these compounds we performed MS analysis. The mass spectrum of compound **8** in aqueous solution showed the presence of the cyclic boronic ester both in the positive ionization mode, as adduct at *m*/*z* 180 ([M + NH_4_]^+^) and *m*/*z* 185 ([M + Na]^+^), and in the negative ionization mode, as *m*/*z* 161 ([M − H]^−^). In the same positive spectrum the presence of a pseudomolecular ion at *m*/*z* 119 revealed the presence of the elimination product **8a** as [M + H]^+^ (C_5_H_10_O_3_) confirming the instability of the β-hydroxyboronic ester. On the other hand, the positive mass spectrum of compound **11** did not reveal the presence of any elimination product, while the negative mass spectrum showed only the pseudomolecular ion at *m*/*z* 145 corresponding to the [M − H]^−^.

The toxicity of compounds **8** and **11**, compared to commercial BPA, on cell viability, was evaluated on human primary fibroblasts. The cells were treated (24–72 h) with increasing concentrations (1–100 μM) of each compound, and cell viability measured by MTT assay. No toxicity was measured at all concentrations and times considered (see Supporting Information File 1), indicating that all compounds under evaluation are biocompatible and suitable for future biological tests.

## Conclusion

In conclusion, we have reported the synthesis, stability evaluation, and preliminary biological testing of two sugar analogues bearing a boron atom at the anomeric position. Compound **8** showed a tendency towards elimination reaction, probably through boronic ester ring opening, while compound **11** demonstrated to be stable. The two compounds showed no toxicity on human primary fibroblasts. Further studies will be conducted to determine the cellular uptake of compounds **8** and **11**.

## Supporting Information

File 1 Experimental procedures and analytical data. Copies of NMR spectra of all new compounds, mass analysis of compounds **8** and **8a** and toxicity data.
