# Supplementary material for: Anomeric sugar boronic acid analogues as potential agents for boron neutron capture therapy
Source: Beilstein J Org Chem. 2019 Jun 19;15:1355–9. doi: 10.3762/bjoc.15.135 (PMC6604725; doi:10.3762/bjoc.15.135)

BEILSTEIN JOURNAL OF ORGANIC CHEMISTRY

Supporting Information

for

Anomeric sugar boronic acid analogues as potential agents for boron neutron capture therapy

Daniela Imperio, Erika Del Grosso, Silvia Fallarini, Grazia Lombardi and Luigi Panza

Beilstein J. Org. Chem. **2019,** *15,* 1355–1359. doi:10.3762/bjoc.15.135

Experimental procedures and analytical data. Copies of NMR spectra of all new compounds, mass analysis of compounds 8 and 8a and toxicity data

License and Terms: This is a supporting information file under the terms of the Creative Commons Attribution License (http://creativecommons.org/licenses/by/4.0). Please note that the reuse, redistribution and reproduction in particular requires that the authors and source are credited.
The license is subject to the *Beilstein Journal of Organic Chemistry* terms and conditions: (https://www.beilstein-journals.org/bjoc)

Table of Contents

Materials and methods S2

Abbreviations S3

Characterization of the products S4

Mass spectral analysis S10

Cytotoxic activities S11

Copies of NMR spectra S12

Materials and Methods

Reagents were used as supplied without further purification unless otherwise stated. All reagents and solvents were purchased from Sigma-Aldrich.

Thin layer chromatography was performed on silica gel plates with fluorescent indicator.

Visualization was accomplished by UV light (254 and/or 365 nm) and/or by staining in ceric ammonium molybdate or sulfuric acid solution.

Anhydrous solvents were obtained using activated molecular sieves (0.3 or 0.4 nm depending on the type of solvent).

All reactions (if not specifically containing water as reactant, solvent or co-solvent) were performed under an argon atmosphere, in oven-dried glassware.

Flash column chromatography was performed following the procedure indicated on *J. Org. Chem*. **1978** *43*, 2923–2925, with 230–400 mesh silica gel.

IR spectra were recorded using a FTIR Thermo-Nicolet Avatar.

NMR spectra were recorded using a JEOL ECP 300 MHz spectrometer. Chemical shifts (δ) are quoted in parts per million referenced to the residual solvent peak. The multiplicity of each signal is designated using the following abbreviations: s, singlet; d, doublet; t, triplet; q, quartet; quint, quintet; sext, sextet; hept, heptet; m, multiplet; brs, broad singlet. Coupling constants (*J*) are reported in hertz (Hz).

Mass spectra were recorded on a Thermo Finningan LCQ-deca XP-plus mass spectrometer equipped with an ESI source and an ion trap detector. Melting points were determined using a Stuart Scientific SMP3 apparatus and remain uncorrected.

Optical rotations were measured on a JASCO P1010 polarimeter at 20 °C.

Abbreviations

BH_3·DMS: borane dimethyl sulfide complex

BH_3·THF: borane tetrahydrofuran complex

DBU: 1,8-diazabicyclo(5.4.0)undec-7-ene

DCM: dichloromethane

DMAP: dimethylaminopyridine

DMF: dimethylformamide

EtOAc: ethyl acetate

MeOH: methanol

PET: petroleum ether

TBAF: tetra-*n*-butylammonium fluoride

THF: tetrahydrofuran

TMSCl: trimethylsilyl chloride

Product characterization

2-*O*-Benzoyl-1,3,4-tri-*O*-benzyl-5-*tert*-butyldiphenylsilylarabinitol (2)

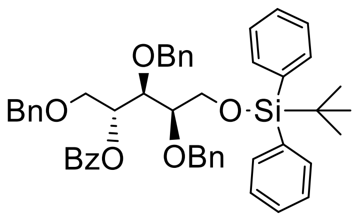

To a solution of **1** (4.00 g, 6.05 mmol) and pyridine (34 mL) in dry CH_2Cl_2 (68 mL) was added dropwise benzoyl chloride (1.4 mL, 12.1 mmol) at 0 °C under argon. The reaction mixture was allowed to warm to room temperature, stirred for 15 h and then worked up by addition of water. The resulting mixture was diluted with CH_2Cl_2 (200 mL), washed with 0.1 N HCl (40 mL), saturated $NaHCO_{3(aq)}$ (40 mL), water (40 mL), and brine (40 mL). The organic layer was dried over $MgSO_4$ and concentrated to give a crude product, which was purified by flash chromatography (hexane/acetone 8:2). The desired product was obtained as yellow oil (4.60 g, 99%).

$[\alpha]^{20}_D$: -7.8 (*c* 1.0, $CHCl_3$).

^{1}H NMR ($CDCl_3$, 300 MHz) δ 8.00 (d, J = 4.6, 2H), 7.70-7.19 (m, 28H), 5.55 (m, 1H), 4.75 (dd, J_1 = 11.3, J_2 = 2.4, 1H), 4.69 (dd, J_1 = 11.6, J_2 = 2.4, 1H), 4.57 (dd, J_1 = 12.0, J_2 = 2.4, 1H), 4.50 (dd, J_1 =9.3, J_2 = 2.7, 1H), 4.43 (dd, J_1 = 8.4, J_2 = 2.7, 1H), 4.34-4.27 (m, 2H), 4.00-3.91 (m, 2H), 3.87 (m, 2H), 3.73 (m, 1H), 1.09 (s, 9H).

^{13}C NMR ($CDCl_3$, 75 MHz) δ 165.7, 138.40, 138.38, 138.2, 135.79, 135.75, 134.7, 133.5, 133.43, 133.1, 130.7, 130.5, 129.89, 129.86, 129.0, 128.5, 128.41, 128.39, 128.31, 128.28, 127.9, 127.8, 127.6, 127.61, 127.59, 100.0, 79.4, 77.4, 74.9, 73.40, 73.38, 73.2, 68.7, 62.8, 27.0, 19.3.

MS (ESI) *m/z* Calculated for $C_{49}H_{52}O_6Si$: 764.35; Found: 787.35 $[M+Na]^+$.

IR (neat, cm^{-1}): 3064, 2929, 2858, 1719, 1270, 1094, 697.

2-*O*-Benzoyl-1,3,4-tri-*O*-benzylarabinitol (3)

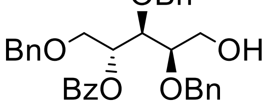

To a stirred solution of TBAF trihydrate (5.51 g, 17.5 mmol) and AcOH (1.12 g, 18.7 mmol) compound **2** was added (4.79 g, 6.26 mmol) as a solution in 31 mL of DMF. After 18 hours the reaction mixture was poured into 20 mL of water and extracted with EtOAc (3 × 20 mL). The combined organic layers were washed with saturated $NaHCO_{3(aq)}$, water, brine and dried over Na_2SO_4. Evaporation under vacuum yielded a crude that was purified by silica gel chromatography (PET/acetone 8:2) to give 2.66 g (80%) of the desired alcohol as yellow oil.

$[\alpha]^{20}_D$: -6.5 (*c* 1.0, $CHCl_3$).

^{1}H NMR ($CDCl_3$, 300 MHz) δ 8.02 (d, *J* = 8.2, 2H), 7.59-7.25 (m, 18H), 5.53 (m, 1H), 4.77 (d, *J* = 11.3, 1H), 4.70 (d, *J* = 11.3, 1H), 4.64-4.50 (m, 4H), 4.11 (t_{app}, *J* = 4.6, 1H), 4.02-3.90 (m, 2H), 3.85-3.74 (m, 3H), 1.97 (bs, 1H).

^{13}C NMR ($CDCl_3$, 75 MHz) δ 165.9, 138.06, 137.99, 133.2, 130.1, 129.9, 128.54, 128.50, 128.47, 128.4, 128.2, 128.0, 127.9, 127.8, 79.3, 78.5, 74.5, 73.5, 73.33, 73.27, 68.3, 61.9.

MS (ESI) *m/z* Calculated for $C_{33}H_{34}O_6$: 526.23 Found: 549.25 $[M+Na]^+$.

IR (neat, cm^{-1}): 3417, 2934, 2878, 1717, 1260, 1088, 1072, 1055, 698.

2-*O*-Benzoyl-1,3,4-tri-*O*-benzyl-5-iodo-5-deoxyarabinitol (4)

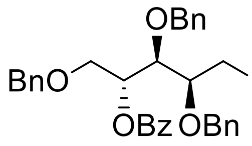

To a vigorously stirred solution of compound **3** (2.66 g, 5.05 mmol) in anhydrous toluene (46 mL) at 70 °C were added triphenylphosphine (1.99 g, 7.57 mmol) and imidazole (1.03 g, 15.15 mmol) under argon. After dissolution, iodine (1.67 g, 6.56 mmol) was added and the heating was continued for 4 h. The solution was cooled and diluted with EtOAc (200 mL). The organic layer was washed with saturated solution of Na_2SO_3 (100 mL), saturated $NaHCO_3$ (100 mL) and brine (100 mL). After drying over sodium sulfate, the solvent was removed under vacuum and the residue purified by column chromatography (PET/acetone 9:1) to yield **4** (3.01 g, 94%) as waxy compound.

$[\alpha]^{20}_D$: +4.4 (*c* 1.0, $CHCl_3$).

^{1}H NMR ($CDCl_3$, 300 MHz) δ 8.02 (d, *J* = 8.2, 2H), 7.59-7.25 (m, 18H), 5.45 (m, 1H), 4.72 (d, *J* = 11.3, 1H), 4.70 (d, *J* = 11.3, 1H), 4.64-4.47 (m, 4H), 4.28 (dd, J_1 = 3.7, J_2 = 6.1, 1H), 3.97 (dd, J_1 = 11.0, J_2 = 3.4, 1H), 3.90-3.81 (m, 2H), 3.35 (m, 2H).

^{13}C NMR ($CDCl_3$, 75 MHz) δ 165.7, 138.0, 137.5, 133.2, 130.1, 129.9, 128.52, 128.47, 128.3, 128.01, 127.97, 127.8, 79.77, 78.15, 75.1, 73.4, 73.3, 68.3, 4.09.

MS (ESI) *m/z* Calculated for $C_{33}H_{33}IO_5$: 636.14; Found: 659.06 $[M+Na]^+$.
IR (neat, cm^{-1}): 3032, 2867, 1717, 1453, 1269, 1094, 1066, 697.

1,3,4-Tri-*O*-benzyl-2-*O*-benzoyl-L-*erhytro*-pent-4-enitol (5)

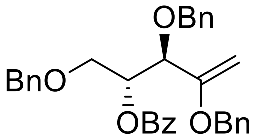

To a stirred solution of compound **4** (4.87 g, 7.65 mmol) in anhydrous toluene (100 mL) was added DBU (2.33 g, 15.30 mmol) and the mixture was heated at refluxed for 4 h under argon. The solution was cooled and evaporated. The residue was dissolved in DCM (50 mL) and washed with water (3 × 100 mL), brine (100 mL) and dried over sodium sulfate. The solvent was removed under vacuum and the residue purified by column chromatography (cyclohexane/acetone 7:3) to yield **5** (2.85 g, 73%) as orange oil.
$[\alpha]^{20}_D$: +2.2 (*c* 1.0, $CHCl_3$).
^1H NMR ($CDCl_3$, 300 MHz) δ 8.02 (d, J = 8.2, 2H), 7.59-7.25 (m, 18H), 5.61 (m, 1H), 4.76-4.39 (m, 7H), 4.27 (m, 2H), 3.94 (m, 2H).
^{13}C NMR ($CDCl_3$, 75 MHz) δ 165.8, 158.2, 138.5, 138.2, 136.9, 133.0, 130.6, 130.0, 129.2, 128.6, 128.4, 128.0, 127.9, 127.7, 127.6, 127.5, 87.0, 78.6, 73.4, 73.3, 71.2, 69.8, 68.7.
MS (ESI) *m/z* Calculated for $C_{33}H_{32}O_5$: 508.22; Found: 531.17 $[M+Na]^+$.
IR (neat, cm^{-1}): 3031, 2869, 1718, 1453, 1268, 1099, 697.

1,3,4-Tri-*O*-benzyl-L-*erhytro*-pent-4-enitol (6)

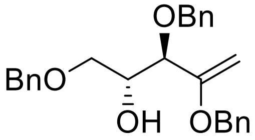

At 0 °C benzoate **5** (2.85 g, 5.60 mmol) was added to a solution 0.6 M of sodium methylate and the resulting reaction mixture was stirred at rt being monitored by TLC. After 24 h, the reaction was evaporated and purified by flash chromatography (cyclohexane/acetone 9:1) to obtain final compound (1.95 g) as yellow oil (yield 86%).
$[\alpha]^{20}_D$: + 45.1 (*c* 1.0, CH_3Cl).

^{1}H NMR ($CDCl_3$, 300 MHz) δ 7.59-7.25 (m, 15H), 4.88 (d, J = 11.9, 1H), 4.83 (d, J = 11.9, 1H), 4.66 (d, J = 11.6, 1H), 4.53 (bs 2H), 4.41 (d, J = 11.6, 1H), 4.35 (bs, 2H), 4.10 (m, 1H), 3.91 (d, J = 6.7, 1H), 3.72 (dd, J_1 = 9.8, J_2 = 3.7, 1H), 3.64 (dd, J_1 = 9.8, J_2 = 5.8, 1H), 2.43 (bs, 1H).

^{13}C NMR ($CDCl_3$, 75 MHz) δ 158.4, 138.4, 138.3, 137.0, 128.7, 128.6, 128.5, 128.1, 128.0, 127.6, 87.3, 80.7, 73.5, 71.2, 71.1, 69.7.

MS (ESI) *m/z* Calculated for $C_{26}H_{28}O_4$: 404.20; Found: 422.06 $[M+18]^+$.

IR (neat, cm^{-1}): 3433, 2924, 2943, 2360, 2340, 1716, 1236, 1093, 698.

(4*S*,5*R*,6*R*)-4,5-bis(benzyloxy)-6-((benzyloxy)methyl)-1,2-oxaborinan-2-ol (7)

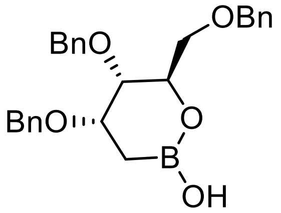

Compound **6** (892 mg, 2.20 mmol) dissolved in dry THF (20 mL) was dropwise added to a solution of BH_3·THF complex (1 M, 11.0 mL, 11.0 mmol) diluted in 12 mL of dry THF at 0 °C under nitrogen. The mixture was stirred for 1 h at room temperature and H_2O (10.0 mL) was slowly added. After stirring for additional 30 h at room temperature, the reaction mixture was evaporated and the residue dissolved in ethyl acetate (20 mL), washed with water (20 mL) and brine (20 mL). The organic layer was dried over sodium sulfate, filtered, and concentrated under vacuum. The crude was purified by flash chromatography (cyclohexane/acetone 9:1) to obtain a colorless oil (305 mg, 32% yield).

$[\alpha]^{20}_{D}$: +36.5 (*c* 1.0, $CHCl_3$).

^{1}H NMR ($CDCl_3$, 300 MHz) δ 7.30 (m, 15H), 4.53 (m, 7H), 3.98 (t, J = 4.9 Hz, 1H), 3.66 (d, J = 7.0 Hz 1H), 3.61 (ddd, J_1 = 5.6 Hz, J_2 = 4.6 Hz, J_3 = 1.8 Hz, 1H), 1.39 (dd, J_1 = 17.0 Hz, J_2 = 5.7 Hz, 1H), 1.04 (dd, J_1 = 17.0 Hz, J_2 = 4.6 Hz, 1H).

^{13}C NMR ($CDCl_3$, 75 MHz) δ 138.9, 138.2, 138.1, 128.5, 128.44, 128.40, 127.9, 127.7, 127.6, 127.5, 76.7, 73.54, 73.50, 72.0, 71.9, 70.9, 70.7, **C**H_2B not detectable.

^{11}B NMR ($CDCl_3$, 96 MHz) δ 29.90.

IR (neat, cm^{-1}): 3425, 3036, 2921, 1453, 1088, 734, 697.

(4*S*,5*S*,6*R*)-6-(hydroxymethyl)-1,2-oxaborinane-2,4,5-triol (8)

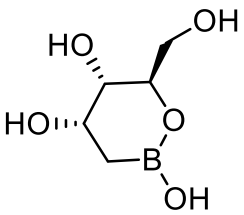

A solution of compound **7** (257 mg, 0.59 mmol) in methanol (8 mL) with 20% $Pd(OH)_2$ on charcoal (97 mg) was stirred under a hydrogen atmosphere. The reaction was complete after 2 h. The suspension was filtered through a Celite pad and the filtrate concentrated under vacuum to afford **8** (94 mg; 98% yield) as colorless wax.

$[\alpha]^{20}_{D}$: +36.5 (*c* 1.0, CH_3OH).

^{1}H NMR (CD_3OD, 300 MHz) δ 4.04 (m, 2H), 3.78-3.62 (m, 3H), 1.04 (d, *J* = 4.9 Hz, 2H).

^{13}C NMR (CD_3OD, 75 MHz) δ 75.5, 69.8, 67.8, 66.1, **C**H_2B not detectable.

^{11}B NMR (CD_3OD, 96 MHz) δ 29.93.

MS (ESI) m/z Calculated for $C_5H_{11}BO_5$: 162.07; Found: 161.13 $[M-1]^-$.

IR (neat, cm^{-1}): 3344, 2921, 1396, 1034, 654.

(4a*S*,8a*R*)-6-Phenylhexahydro-2*H*-[1,3]dioxino[4,5-*e*][1,2]oxaborinin-2-ol (10)

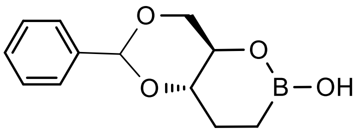

Compound **9** (100 mg, 0.48 mmol) dissolved in dry THF (1.5 mL) was added dropwise to a solution of BH_3·DMS (0.23 mL, 2.4 mmol) at 0 °C under argon. The mixture was stirred for 2 h at room temperature and H_2O (2.0 mL) was slowly added. After stirring for additional 3 h at room temperature, the reaction mixture was diluted with ethyl acetate (20 mL), washed with water (20 mL) and brine (20 mL). The organic layer was dried over sodium sulfate, filtered, and concentrated. The crude was purified by flash chromatography (cyclohexane/ethanol 95:5) to obtain a white solid (45 mg, 49% yield).

Mp: 71-73 °C. $[\alpha]^{20}_{D}$: -6.5 (*c* 1.0, $CHCl_3$).

^{1}H NMR (CD_3OD, 300 MHz) δ 7.35 (m, 5H), 5.54 (s, 1H), 4.26 (dd, 1H, J_1 = 10.4 Hz, J_2 = 4.9 Hz), 3.78 (bs, 1H), 3.62 (t_{app} 1H, J = 10.4 Hz), 3.50 (ddd, 1H, J_1 = 10.7 Hz, J_2 = 4.9 Hz, J_3 = 4.6 Hz), 1.94 (bs, 1H), 1.64 (bs, 1H), 1.08 (m, 1H), 0.90 (m, 1H).

^{13}C NMR (CD_3OD, 75 MHz) δ 138.1, 128.6, 127.8, 126.1, 101.5, 79.9, 70.5, 69.9, 25.1, **C**H_2B not detectable.

^{11}B NMR (CD_3OD, 96 MHz) δ 30.08.

MS (ESI) *m/z* Calculated for $C_{12}H_{15}BO_4$: 234.1; Found: 235.1 $[M+1]^+$.

IR (neat, cm^{-1}): 3448, 2940, 2872, 1383, 1338, 1014, 752.

(5*S*,6*R*)-6-(hydroxymethyl)-1,2-oxaborinane-2,5-diol (11)

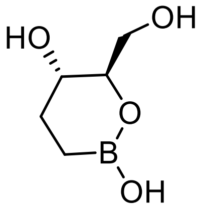

A solution of compound **10** (130 mg, 0.55 mmol) in methanol (6 mL) with a spoon tip of 10% $Pd(OH)_2$ on charcoal was stirred under a hydrogen atmosphere. The reaction was complete after 24 h. The suspension was filtered and the solution concentrated under vacuum to afford **11** (80 mg; 99% yield) as white wax.

$[\alpha]^{20}_D$: + 15.8 (*c* 1.0, CH_3OH).

^{1}H NMR (CD_3OD, 300 MHz) δ 3.67 (m, 4H), 1.87 (bs, 1H), 1.58 (m, 1H), 0.95 (m, 1H), 0.74 (m, 1H,).

^{13}C NMR (CD_3OD, 75 MHz) δ 79.8, 67.5, 63.3, 26.8, **C**H_2B not detectable.

^{11}B NMR (CD_3OD, 96 MHz) δ 29.63.

MS (ESI) *m/z* Calculated for $C_5H_{11}BO_4$: 146.1; Found: 145.2 $[M-]^-$.

IR (neat, cm^{-1}): 3344, 2929, 1675, 1369, 1231, 1186, 1027, 722.

MS analysis of compounds 8 and 8a

All mass spectra were recorded with a Thermo Finnigan LCQ Deca XP Plus equipped with an ESI source in water solution. The data recorded in positive ionization mode (Figure S1) demonstrated the presence in water solution of **8a** ($C_5H_{10}O_3$) at *m*/*z* 119 and compound **8** ($C_5H_{11}BO_5$) as adduct. In the negative ionization mode (Figure S2) only compound **8** is present at *m*/*z* 161 $[M{-}H]^-$.

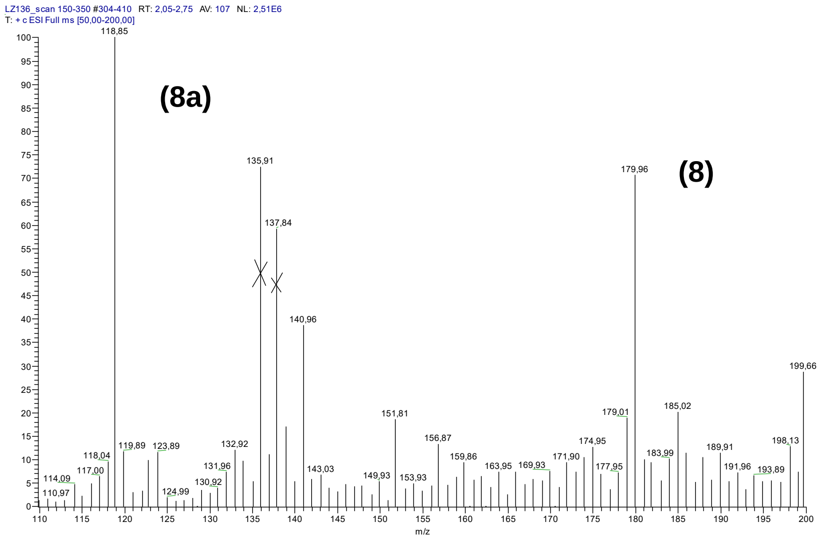

Figure S1 Full (+) MS: *m*/*z* 119 $[M + H]^+$ corresponding to **8a**; *m*/*z* 180 $[M + NH_4]^+$ corresponding to **8**.

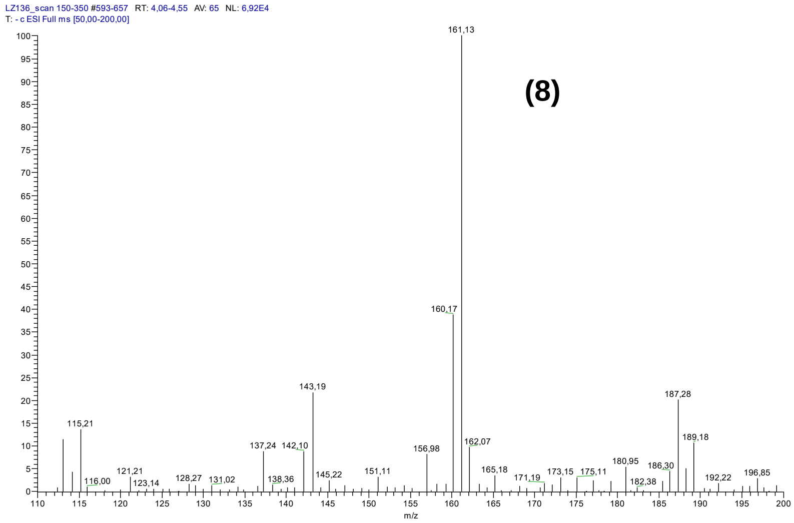

Figure S2 Full (−) MS: *m*/*z* 161 $[M{-}H]^-$ corresponding to **8**.

Toxicity testing

Cell culture

Human primary fibroblasts (ATCC; American Type Culture Collection, Manassas, VA, USA) were isolated from abdominal skin biopsy of a healthy 52-year old woman. Fibroblasts were cultured in fibroblast basal medium supplemented with 2% fetal bovine serum (FBS), 5 ng/mL recombinant human (rh) fibroblast growth factor (FGF) b, 7.5 mM L-glutamine, 50 μg/mL ascorbic acid, 1 μg/mL hydrocortisone hemisuccinate, 5 μg/mL rh insulin, and cultured in a humidified atmosphere (5% CO_2, 37 °C). Cells were growth in 25 cm^2 culture flasks and sub-cultured when they reached 80% confluence.

Cell viability assay

Cell viability was measured by the 3-(4,5-dimethylthiazol-2-yl)-2,5- diphenyl-tetrazolium bromide (MTT) assay, as previously described. Fibroblasts were seeded (5 × 10^3 cells/ well) in 96-well plates in complete medium. After 24 h of cell attachment, fibroblasts were treated with increasing concentrations (1–100 μM) of test compounds for 24–72 h at 37 °C in a 5% CO_2 humidified incubator. The percentage of cell viability was calculated as [100 (x−y)/(z−y)], where x, y, and z were the absorbances read in compound-treated, resting, and compound-untreated cells, respectively. Results are expressed as mean ± SEM. of at least three experiments run in triplicate.

Results

To evaluate the effect of compounds **8**, **11** and BPA on cell viability, the human primary fibroblasts were used. Cells were treated (24–72 h) with increasing concentrations (1–100 μM) of each compound, and cell viability measured by MTT assay. No toxicity was measured at all concentrations and times considered (Figure S3), indicating that all of compounds under evaluation are biocompatible and suitable for future biological tests.

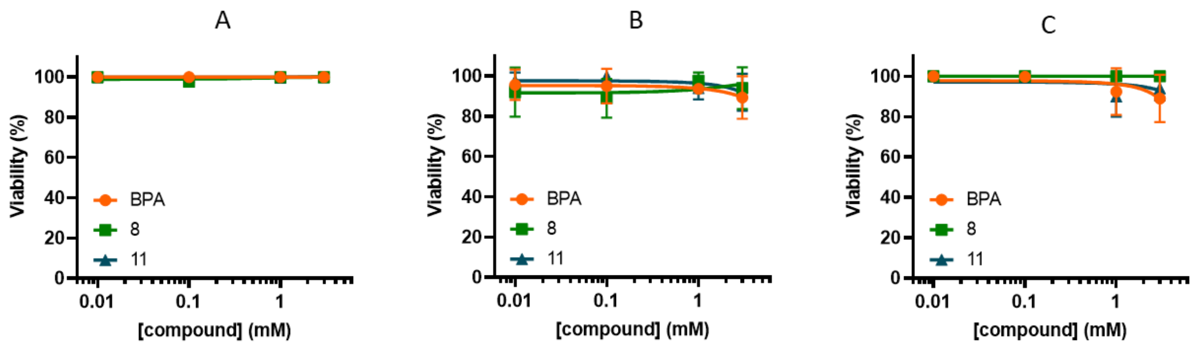

Figure S3. Effects of compounds **8**, **11**, and boronophenylalanine (BPA) on cell viability. Human primary fibroblasts were treated with increasing concentrations (1–100 μM) each compound for 24 h (A), 48 h (B) and 72 h (C). Cell viability was assessed by MTT assay. The concentration–response curves show the percentage of cell viability in comparison with controls (untreated cells). The data represent mean ± SEM of at least three independent experiments run in triplicate.

^{1}H NMR (301 MHz, CHLOROFORM-D)

(2)

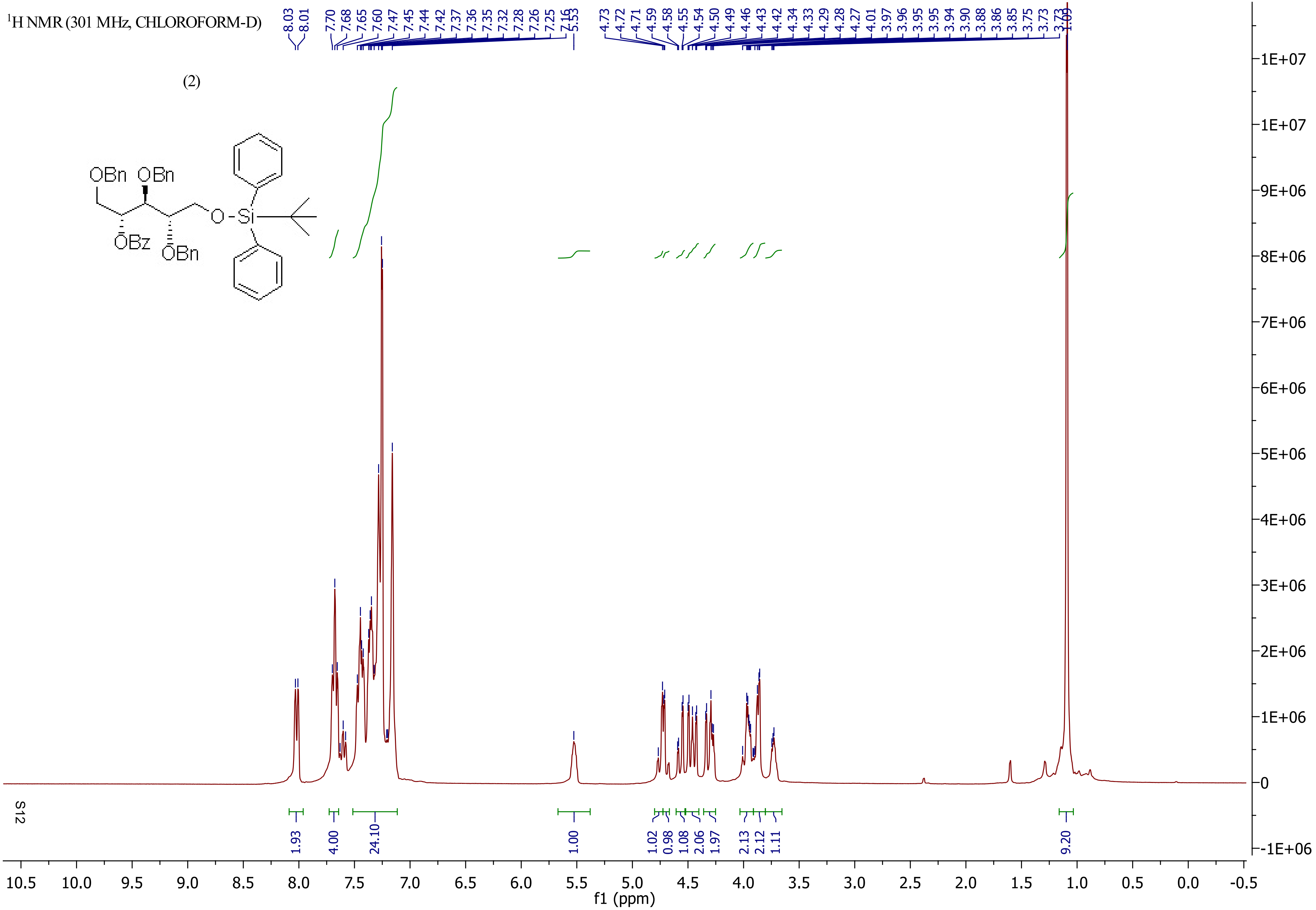

^{13}C NMR (76 MHz, CHLOROFORM-D)

(2)

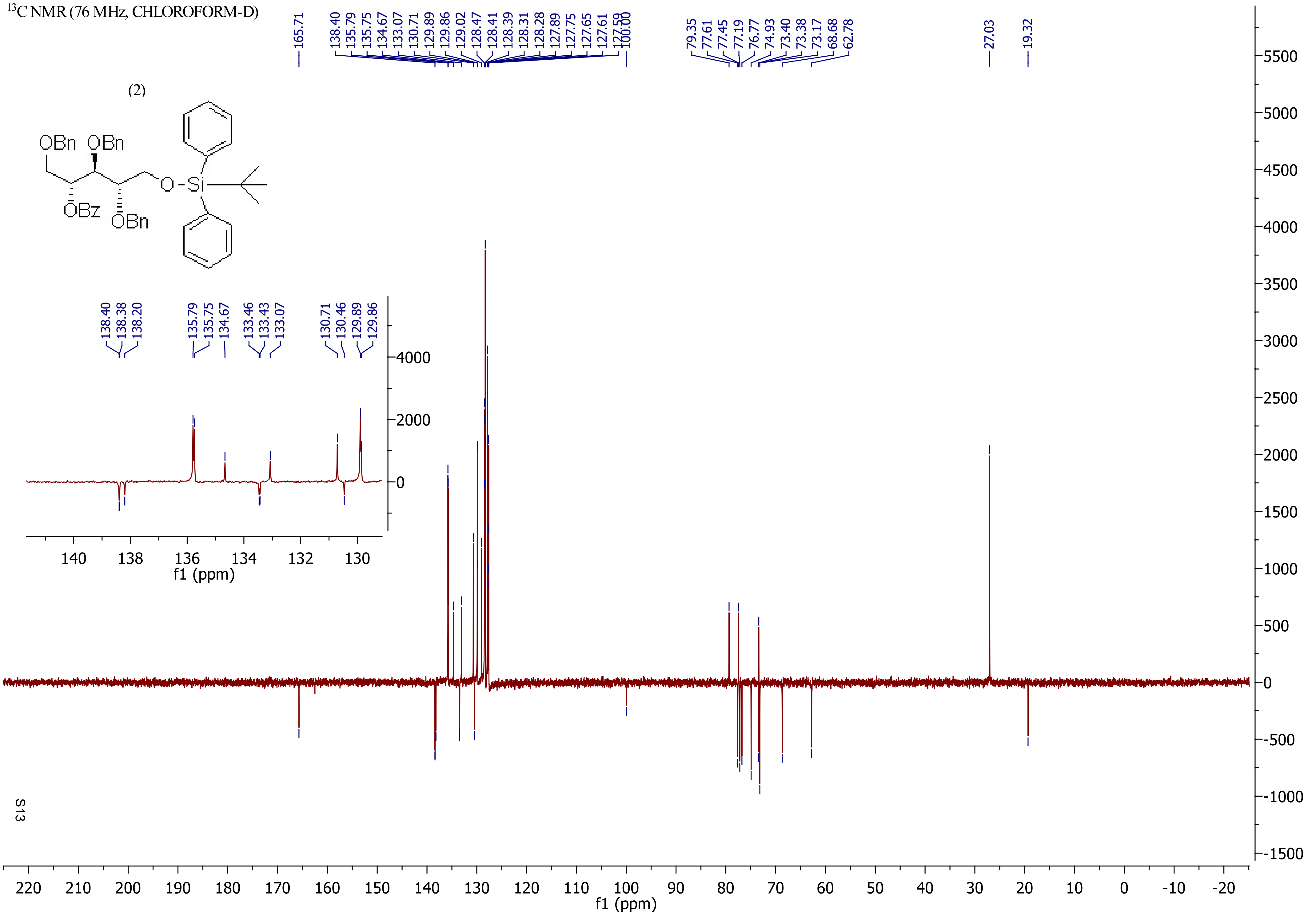

^{1}H NMR (301 MHz, CHLOROFORM-D)

(3)

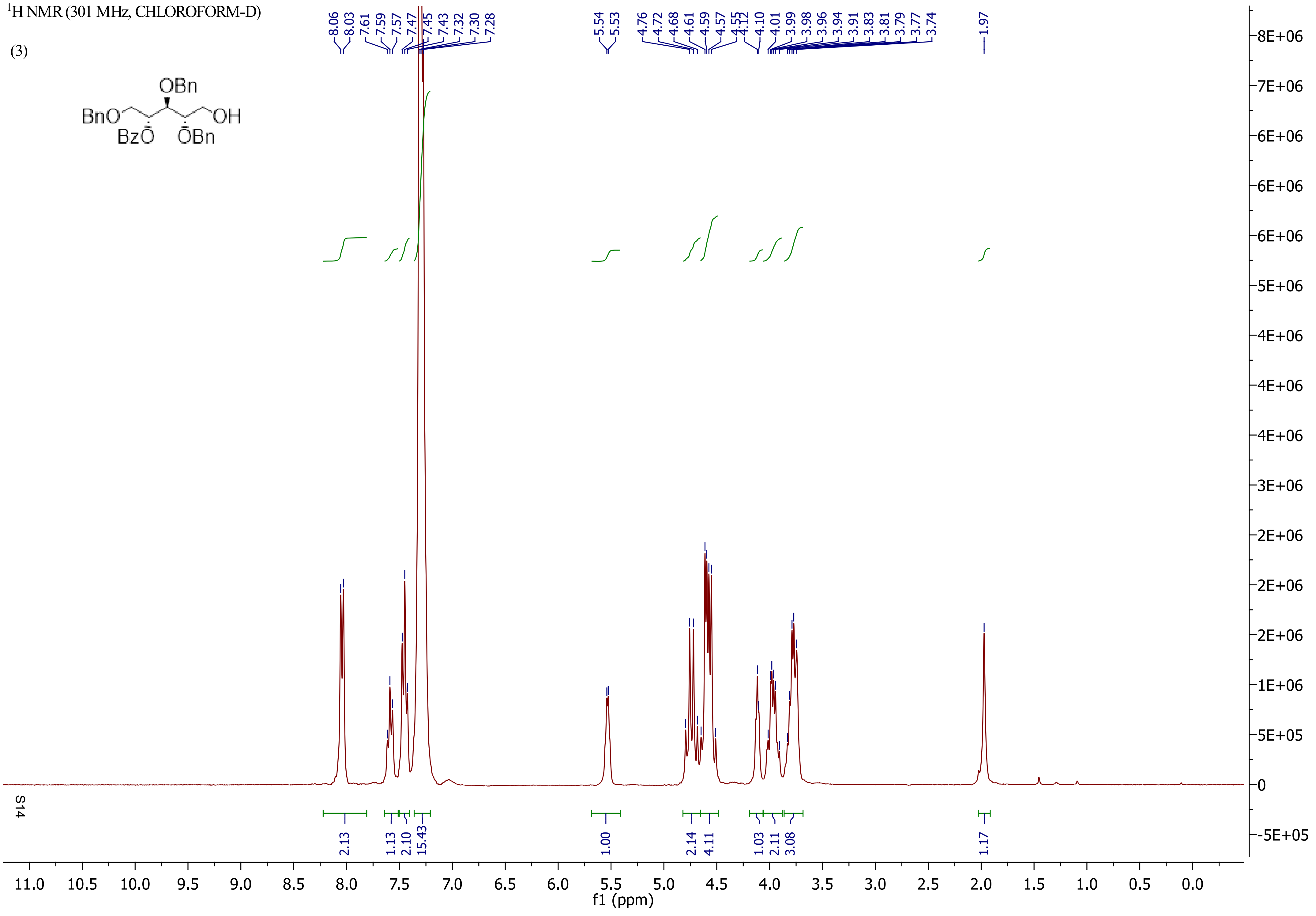

^{13}C NMR (76 MHz, CHLOROFORM-D)

(3)

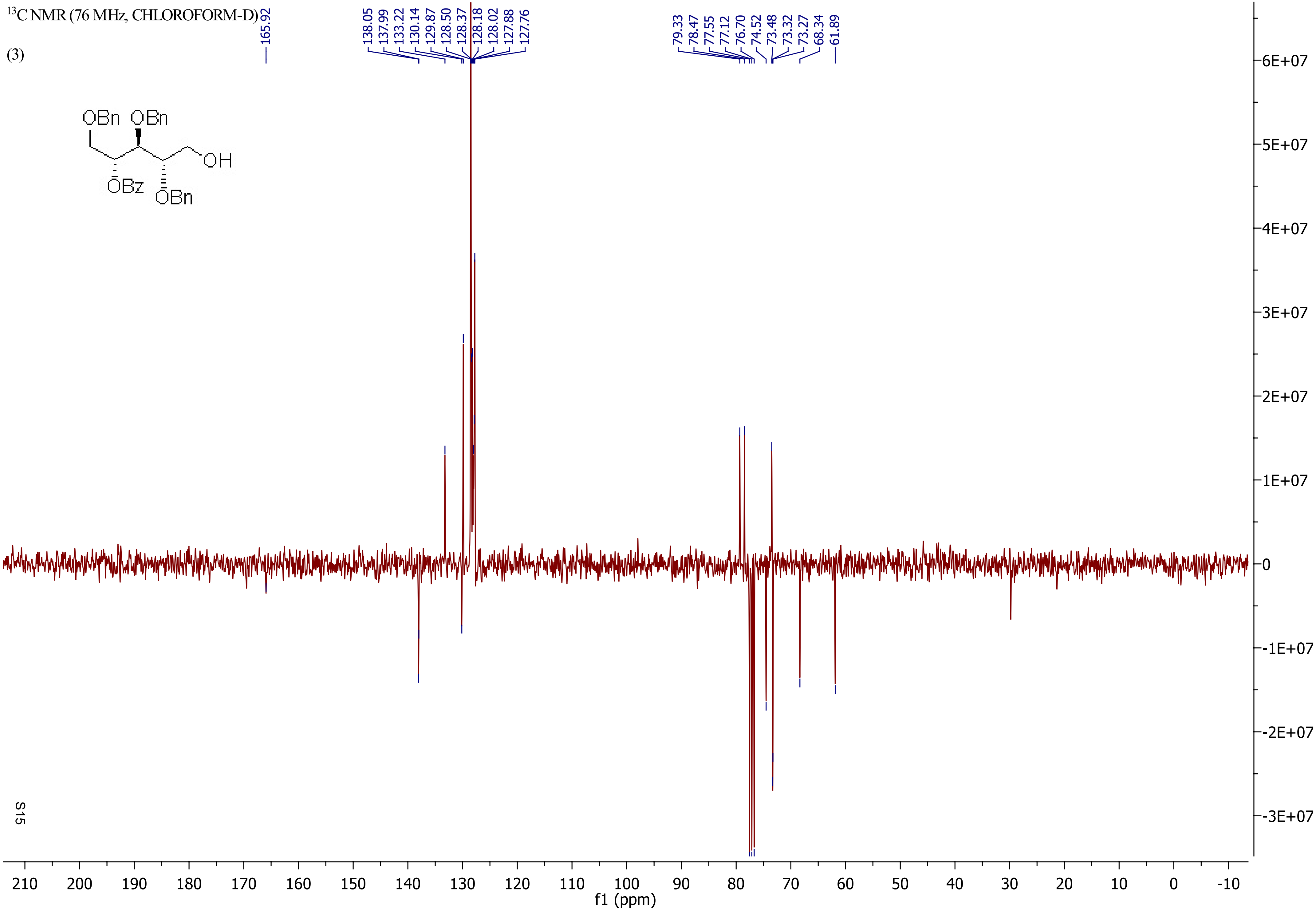

^{1}H NMR (301 MHz, CHLOROFORM-D)

(4)

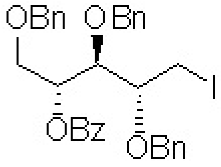

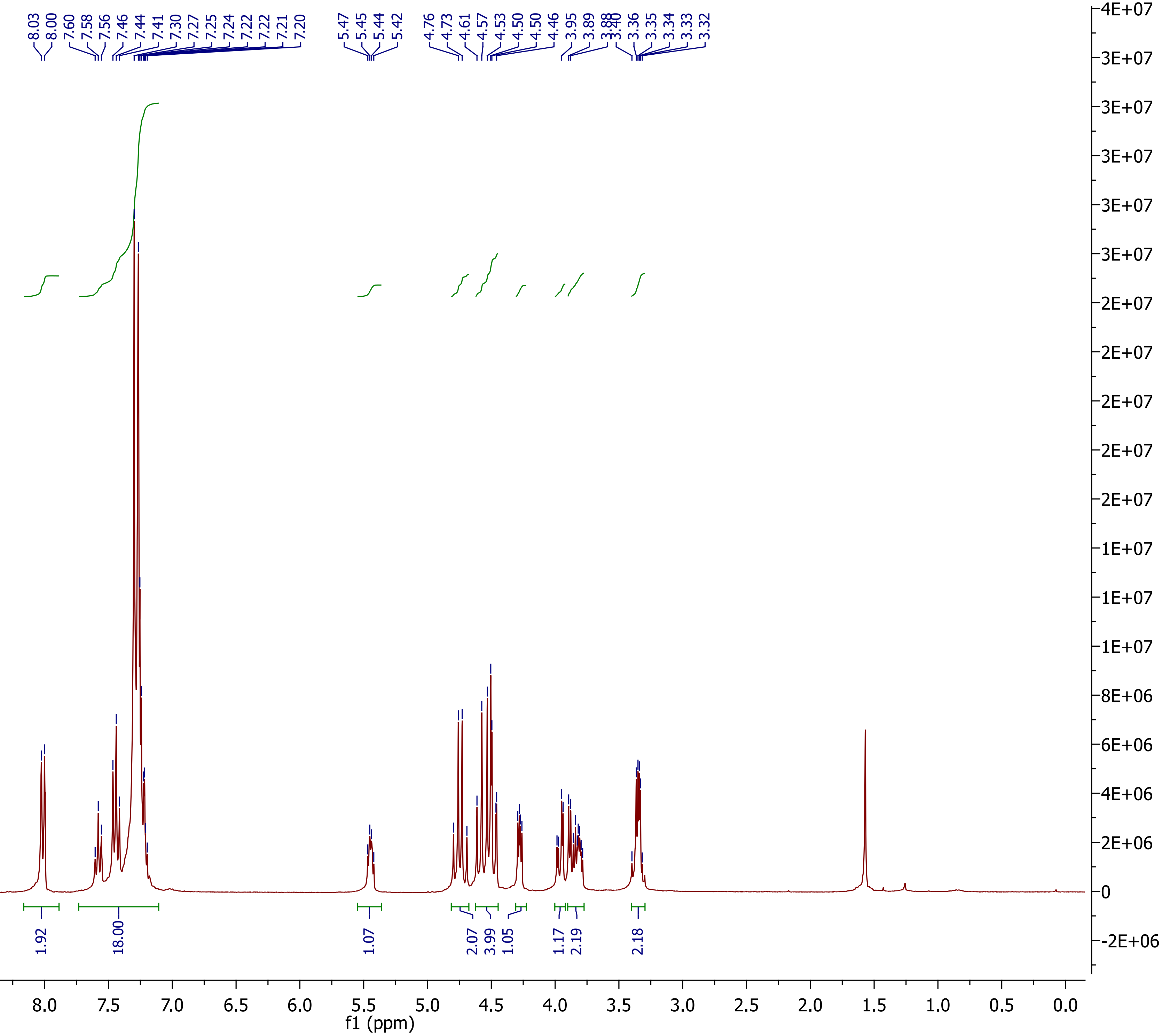

S16

^{13}C NMR (76 MHz, CHLOROFORM-D)

(4)

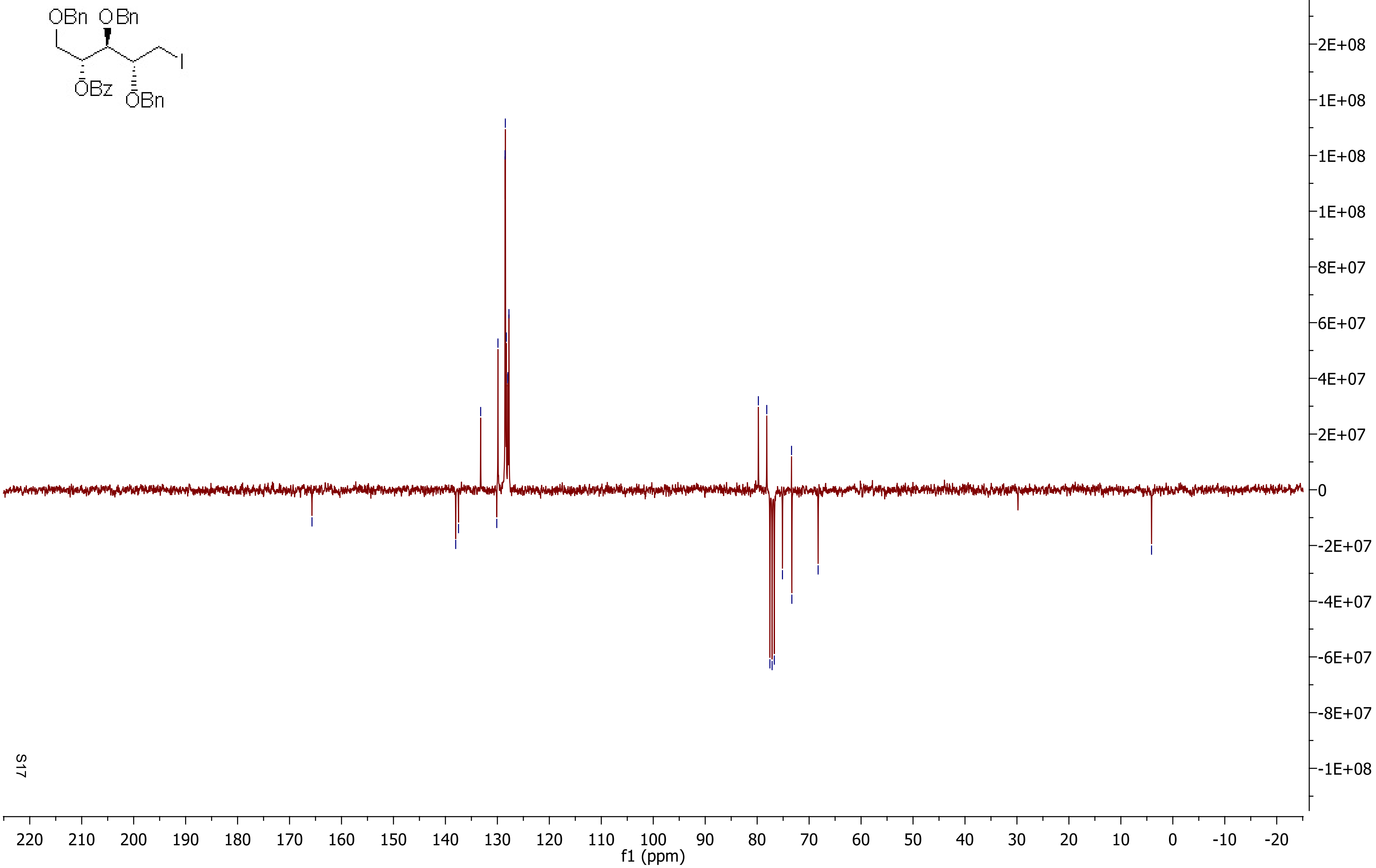

^{1}H NMR (301 MHz, CHLOROFORM-D)

(5)

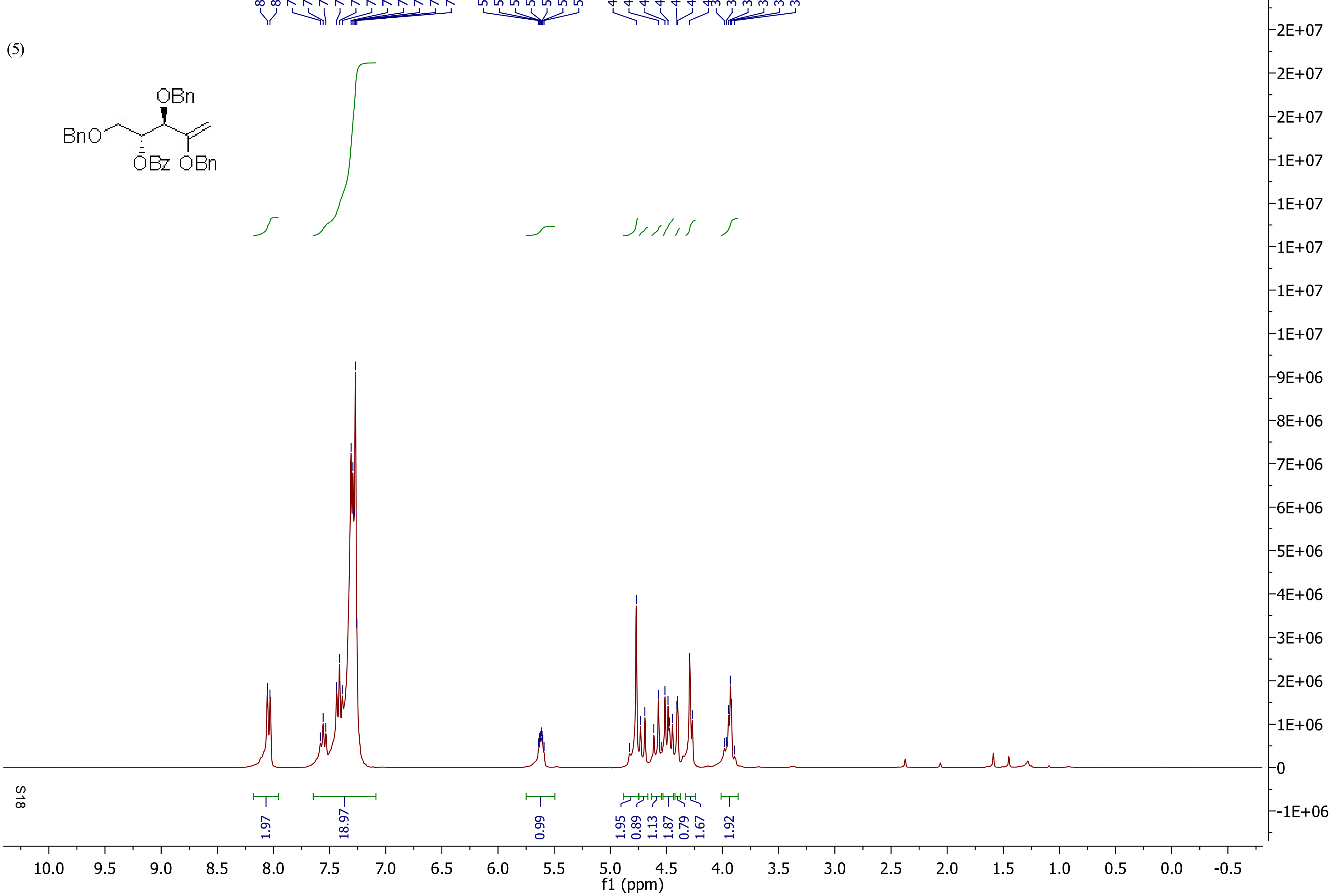

^{13}C NMR (76 MHz, CHLOROFORM-D) δ 165.77, 158.06, 138.36, 138.07, 136.82, 132.95, 130.50, 129.95, 128.49, 128.39, 127.92, 127.81, 127.68, 127.59, 127.46, 86.96, 78.49, 77.58, 77.16, 76.74, 73.27, 73.19, 71.16, 69.73, 68.56

(5)

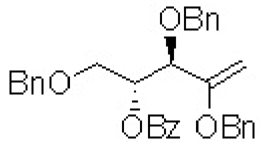

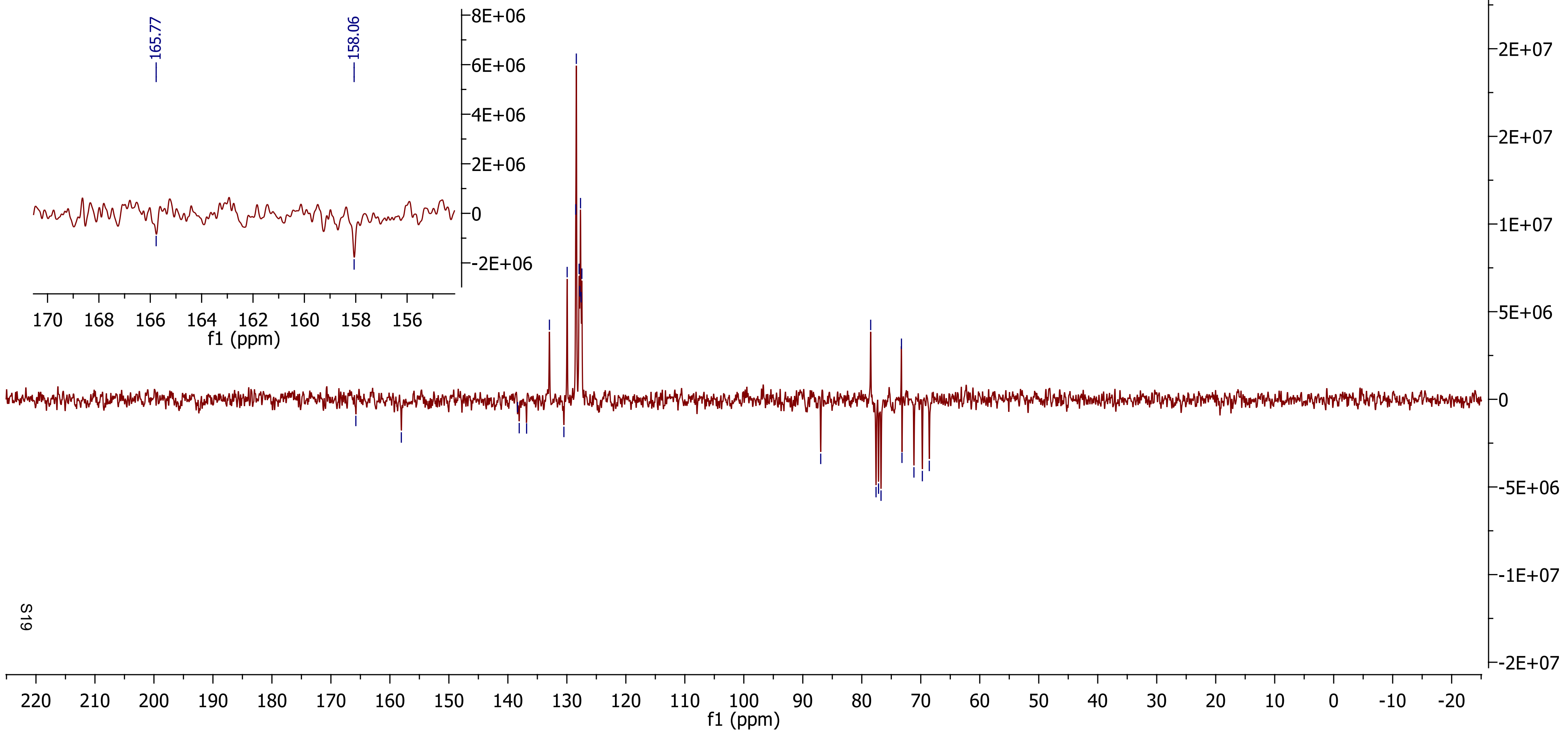

^{1}H NMR (301 MHz, CHLOROFORM-D)

(6)

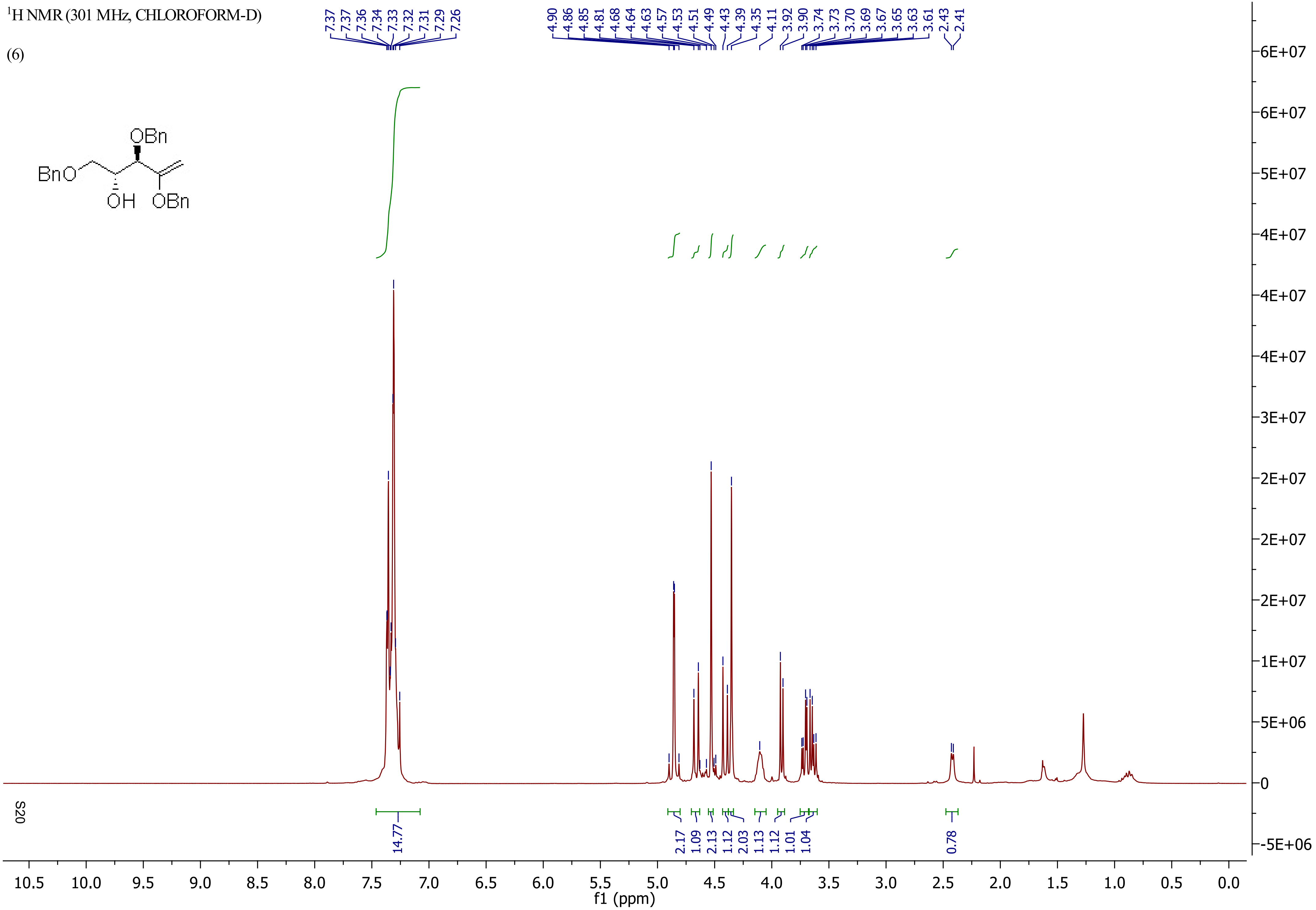

^{13}C NMR (76 MHz, CHLOROFORM-D)

(6)

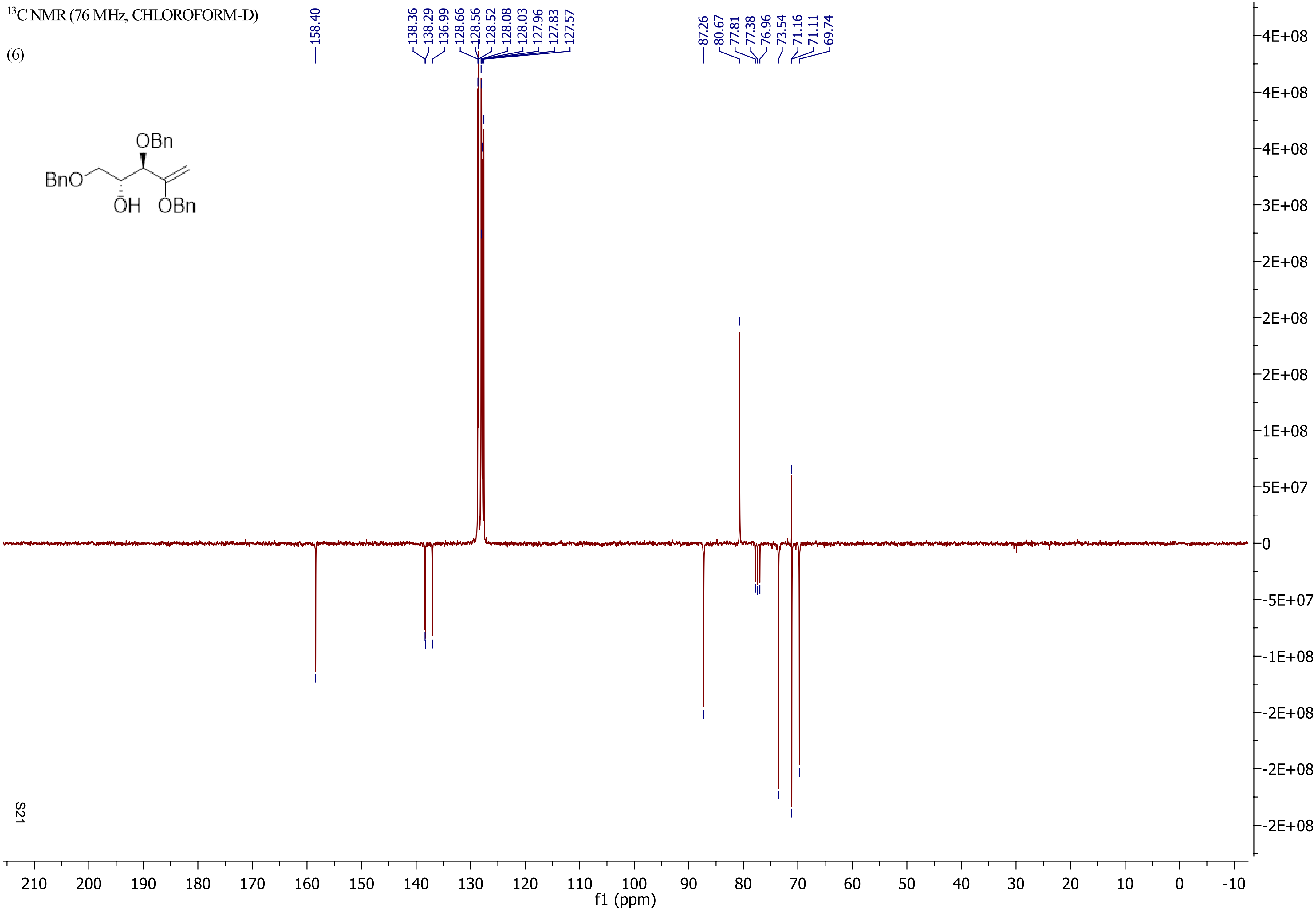

^{1}H NMR (301 MHz, CHLOROFORM-D)

(7)

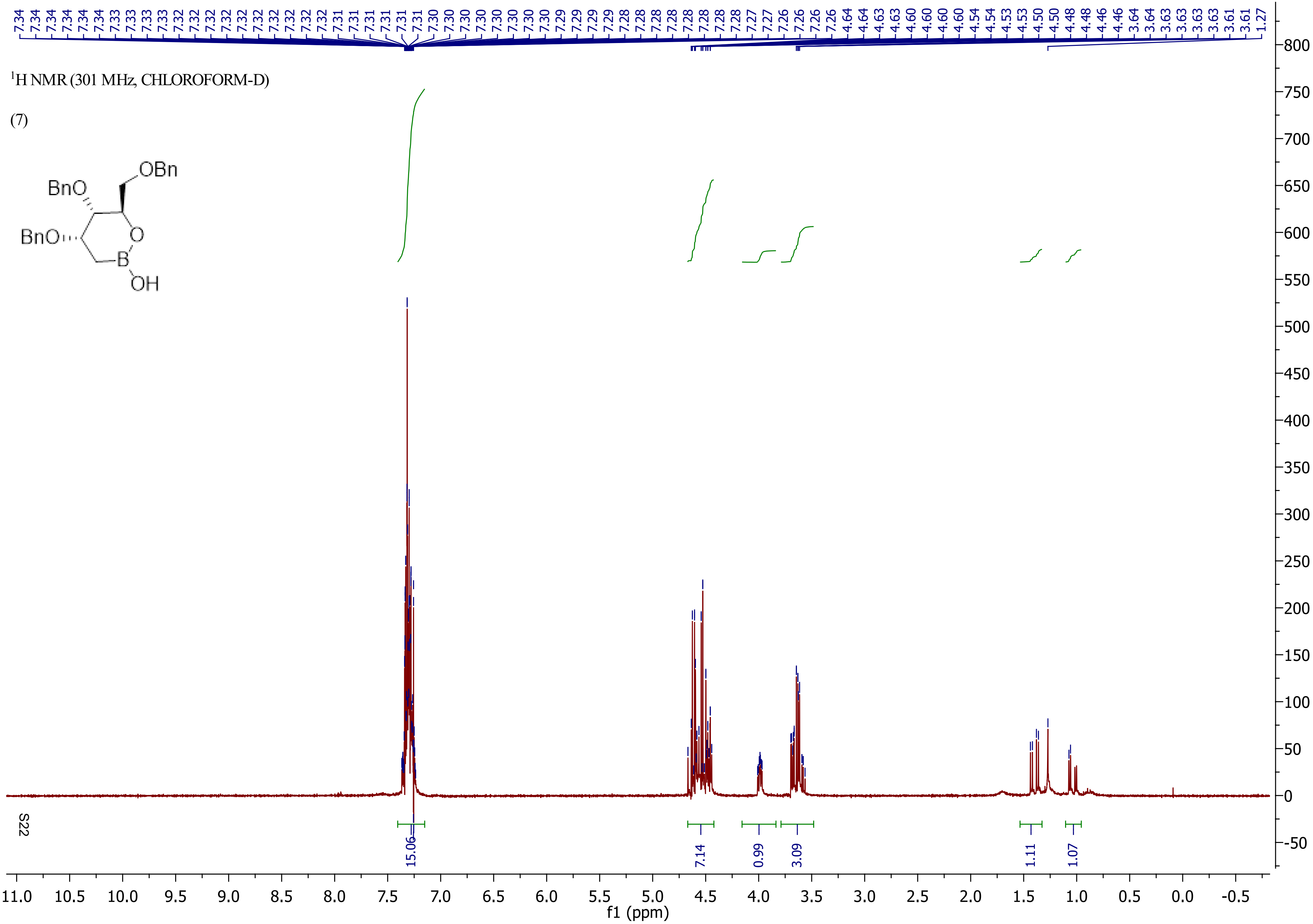

^{13}C NMR (76 MHz, CHLOROFORM-D)

(7)

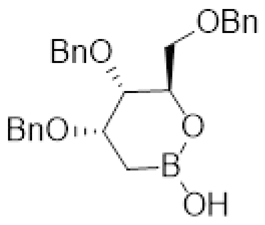

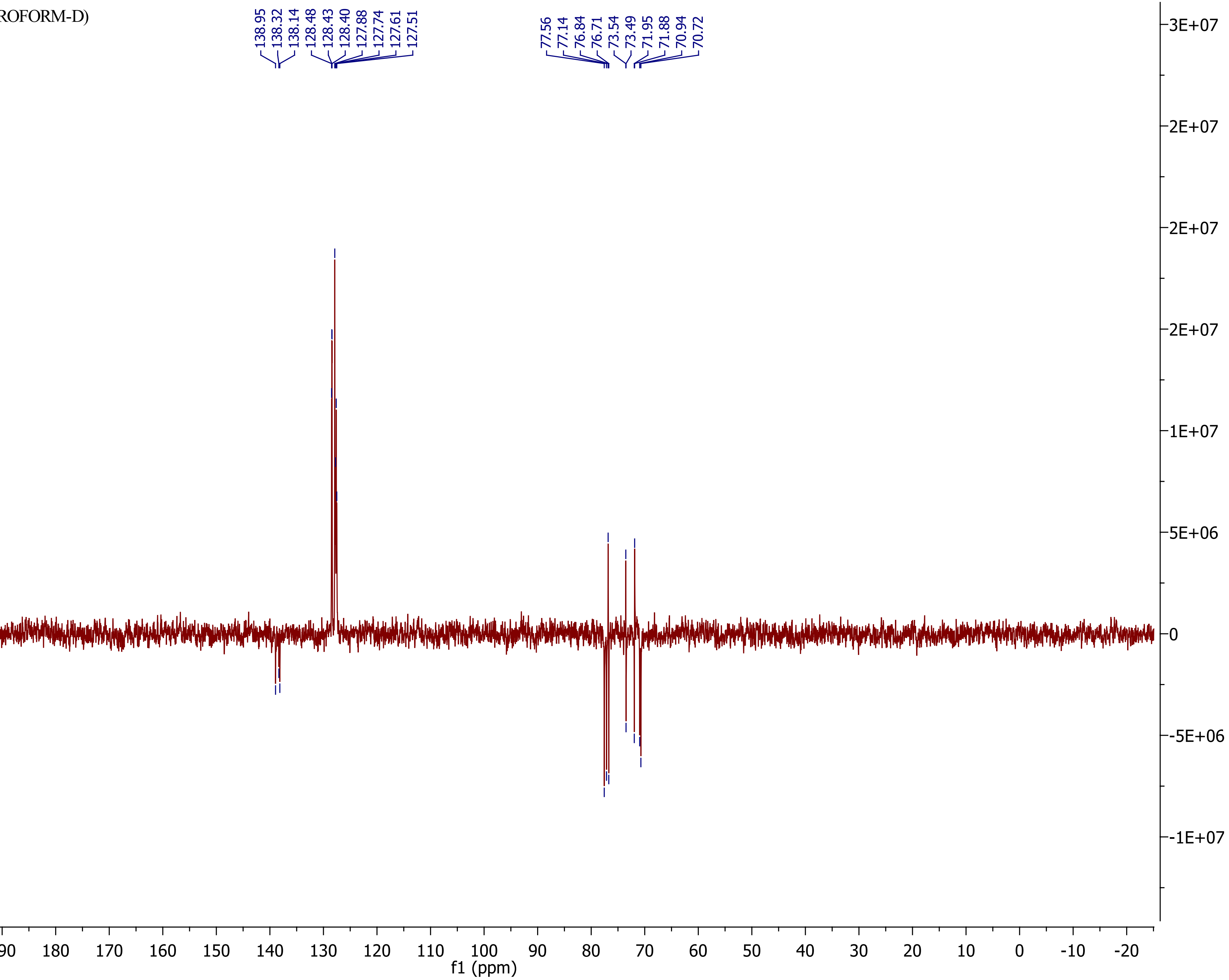

(7) Cosy

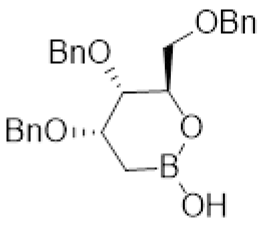

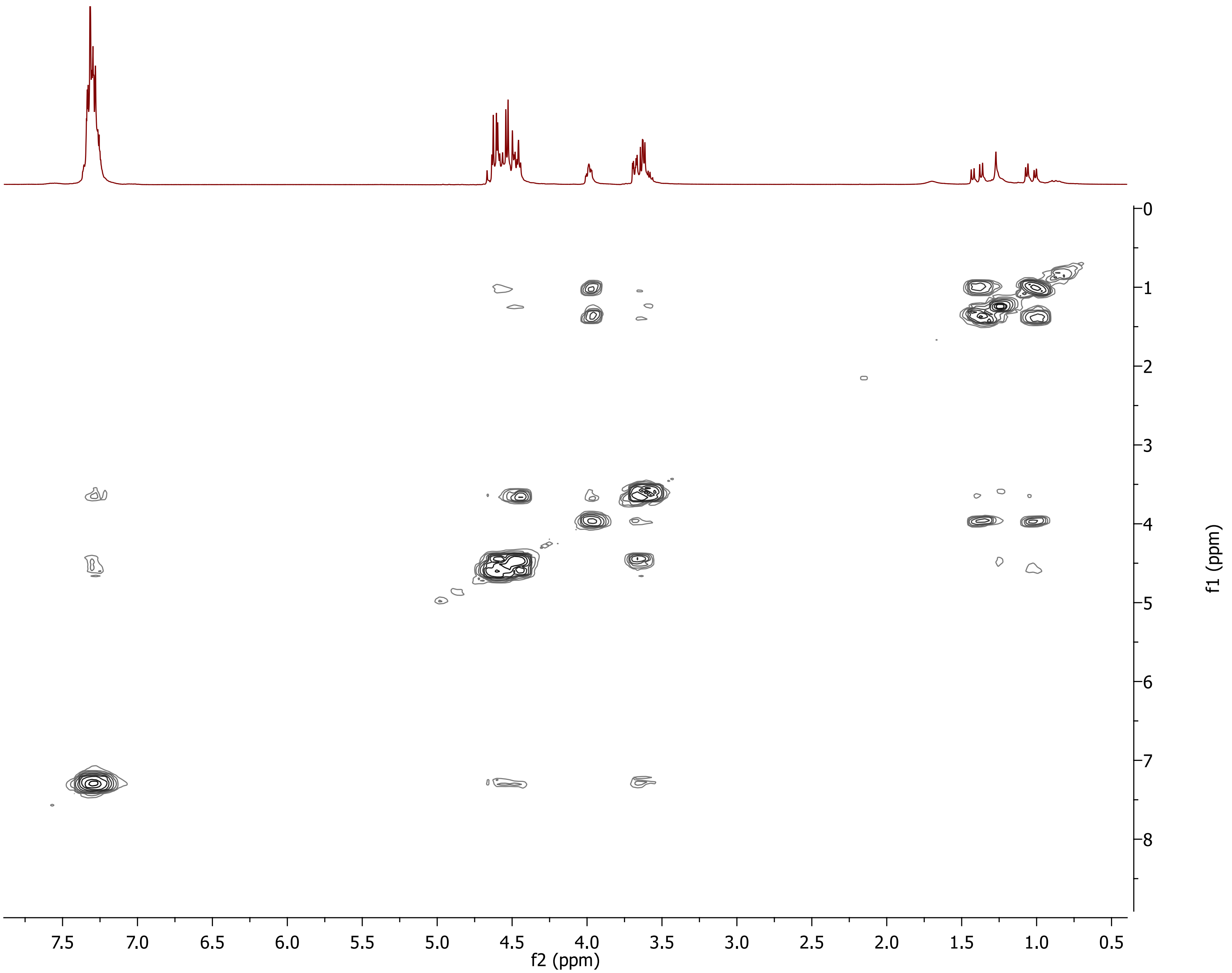

^{11}B NMR (96 MHz, CHLOROFORM-D)

(7)

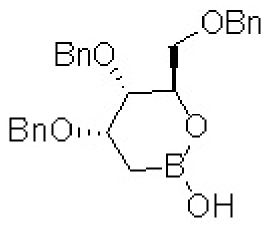

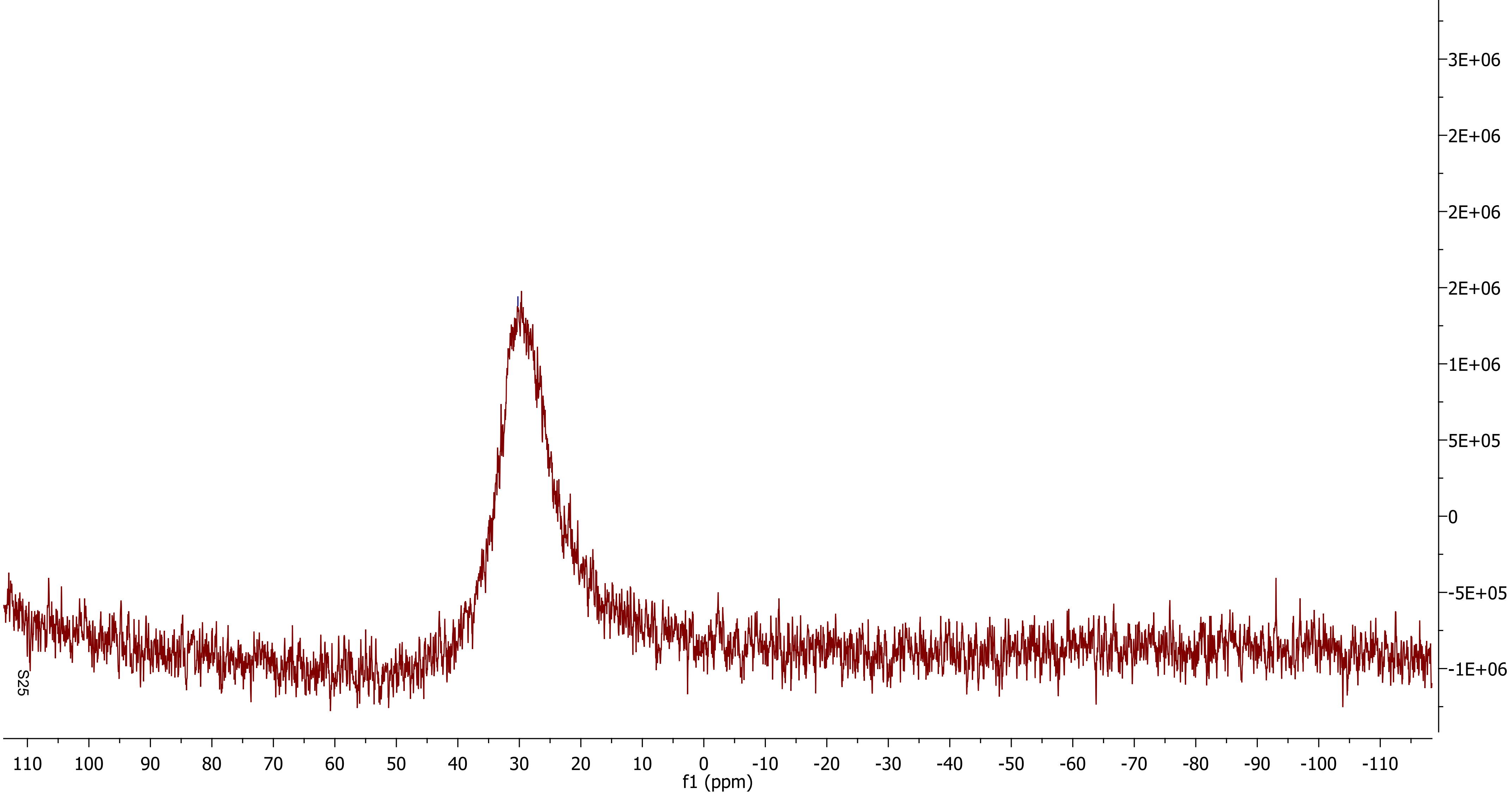

^{1}H NMR (301 MHz, METHANOL-D3)

(8)

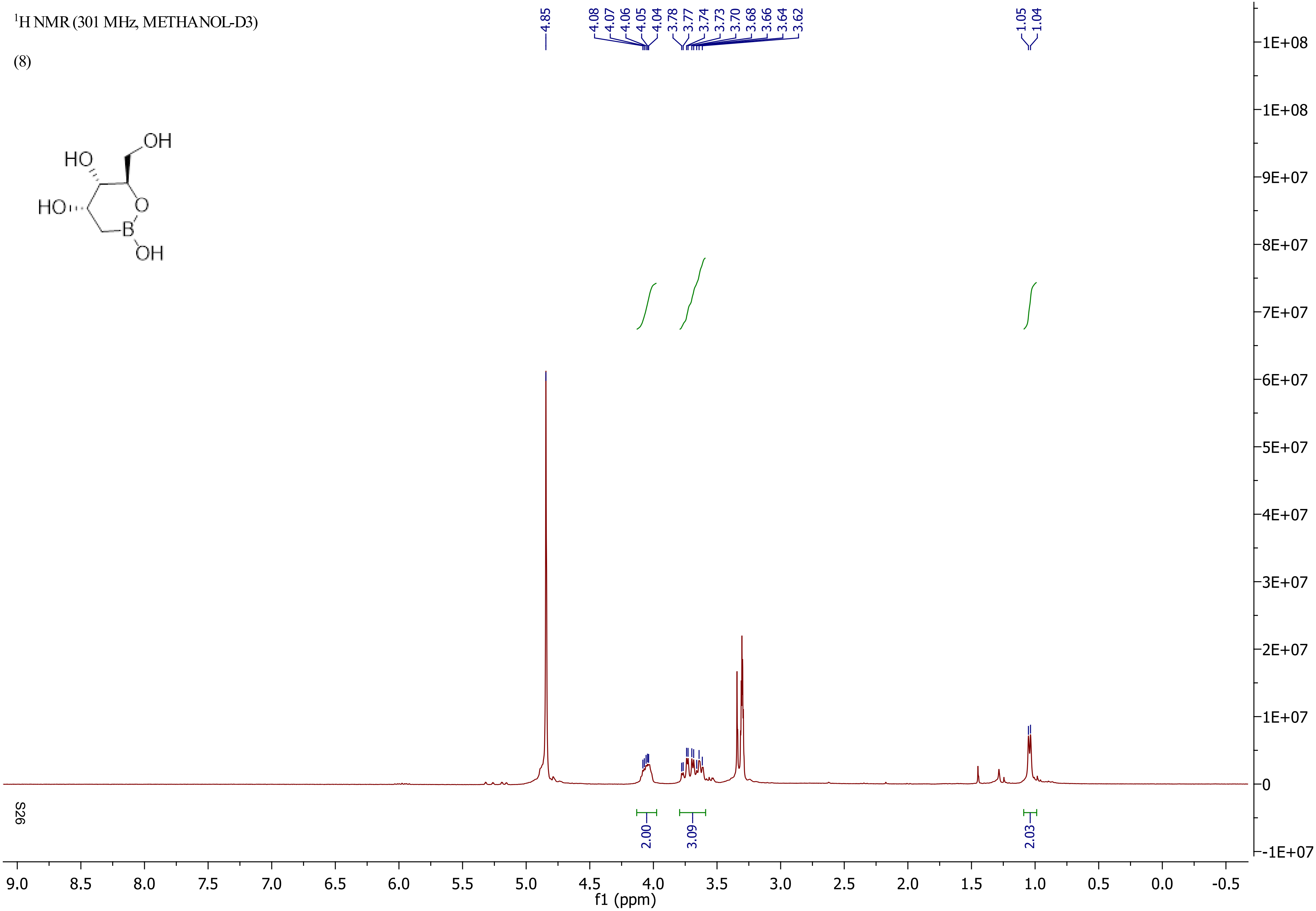

^{13}C NMR (76 MHz, METHANOL-D3)

(8)

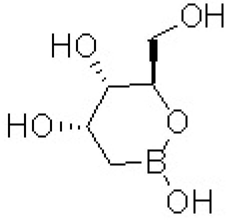

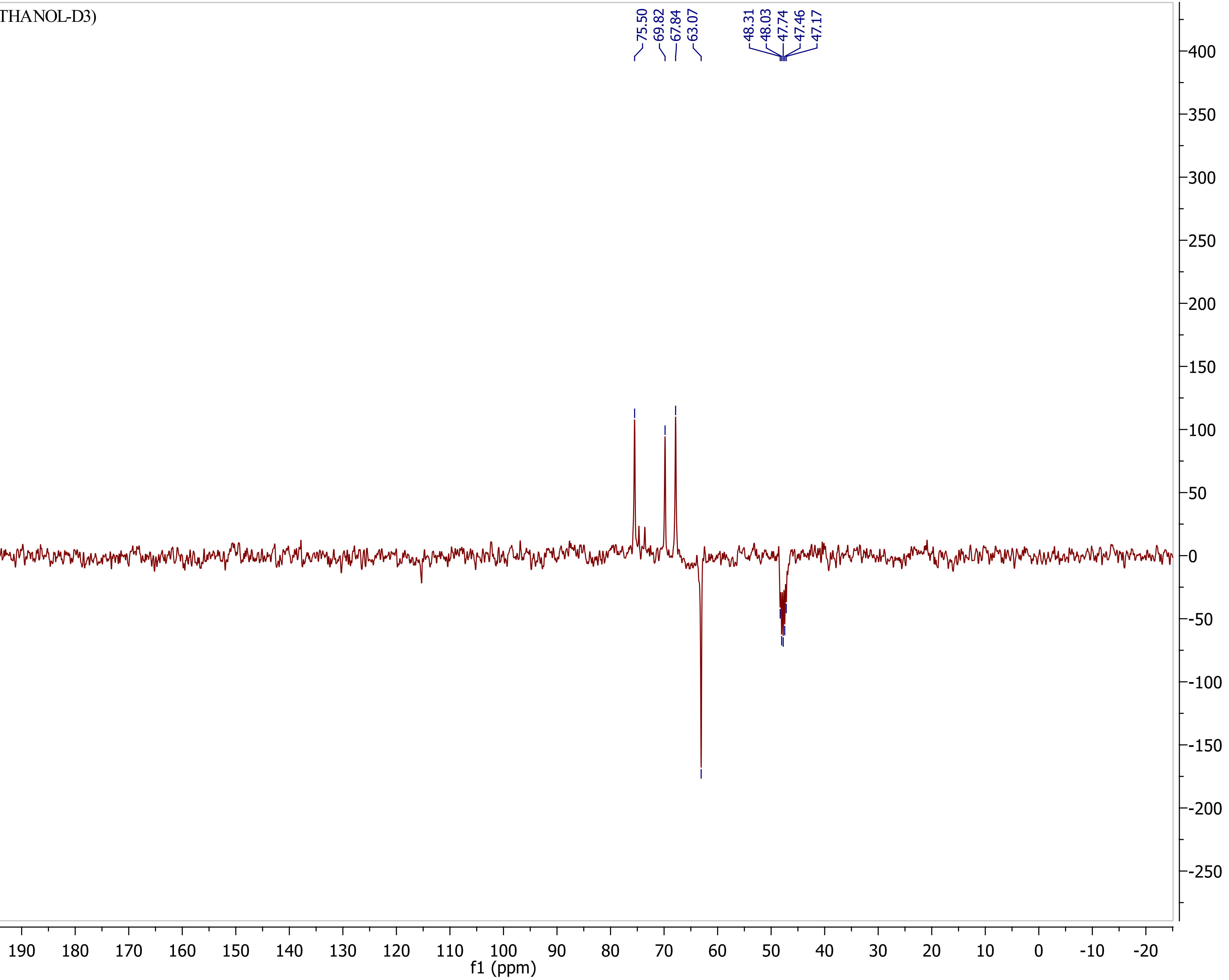

^{11}B NMR (96 MHz, METHANOL-D3)

(8)

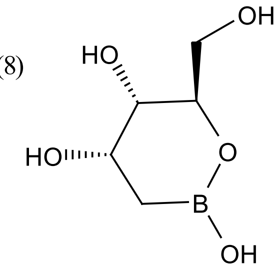

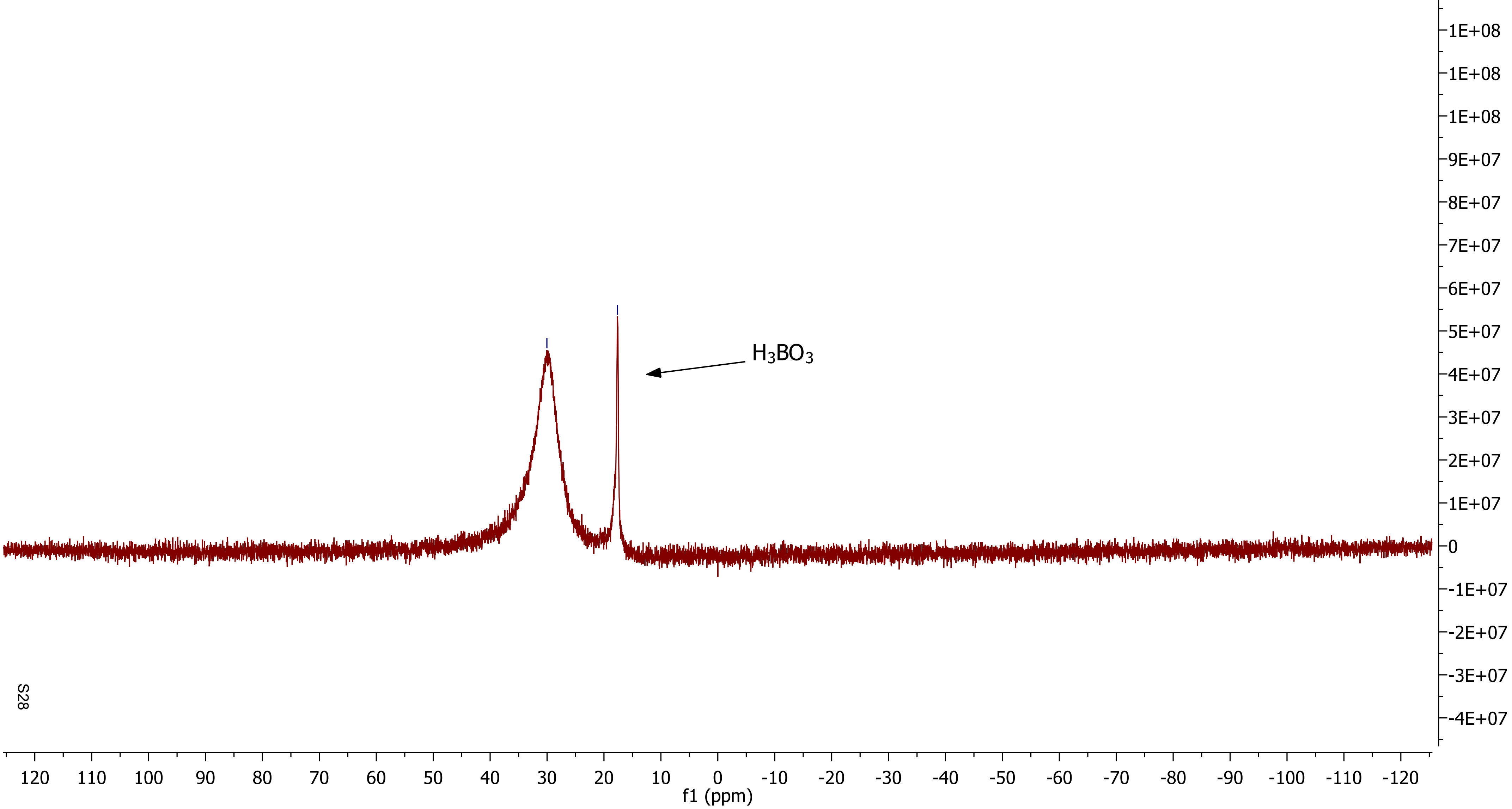

^{1}H NMR (301 MHz, METHANOL-D3)

(10)

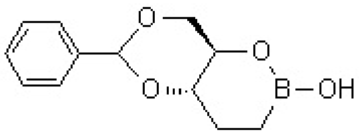

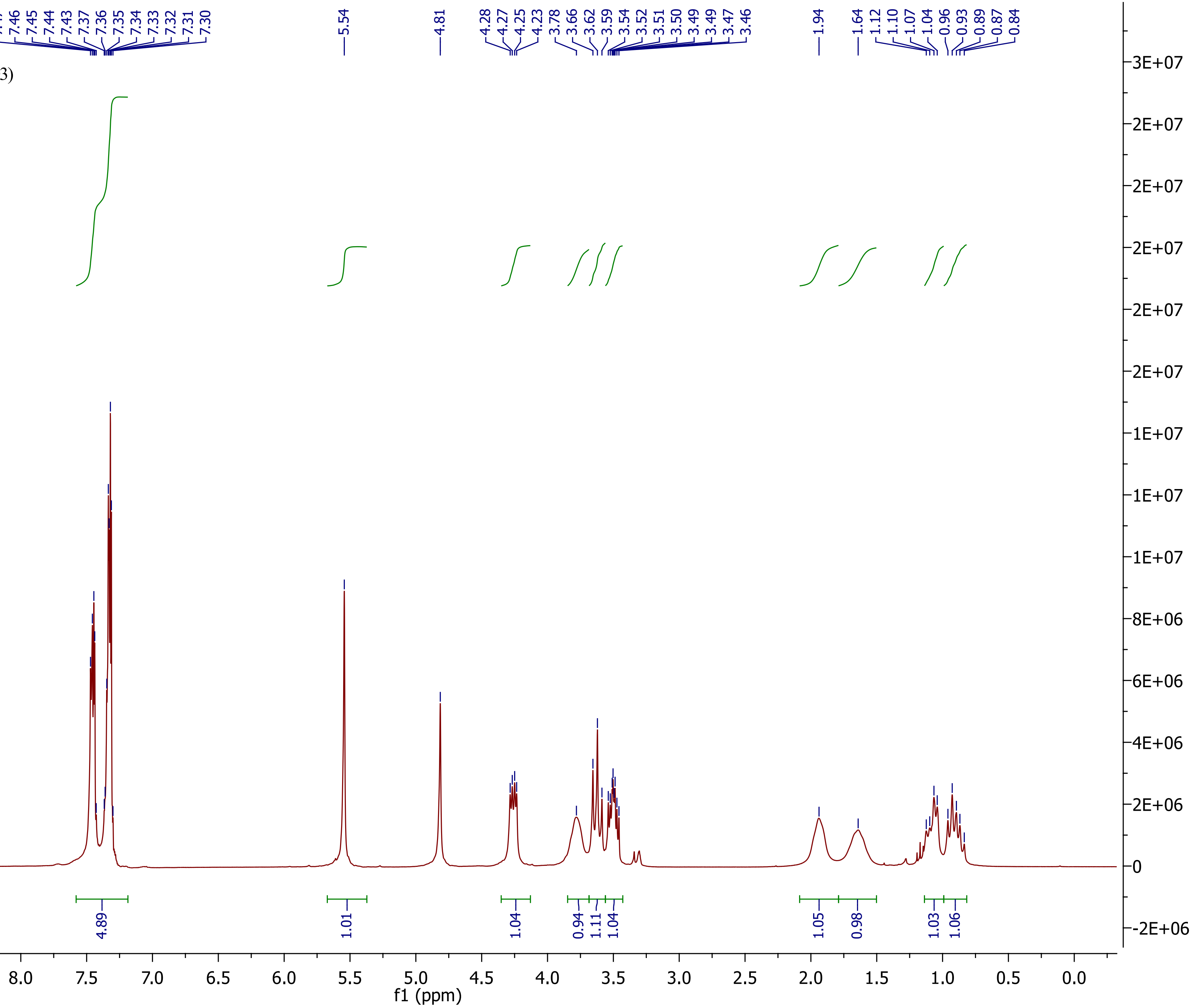

^{13}C NMR (75 MHz, METHANOL-D3)

(10)

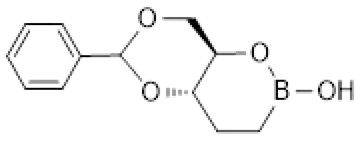

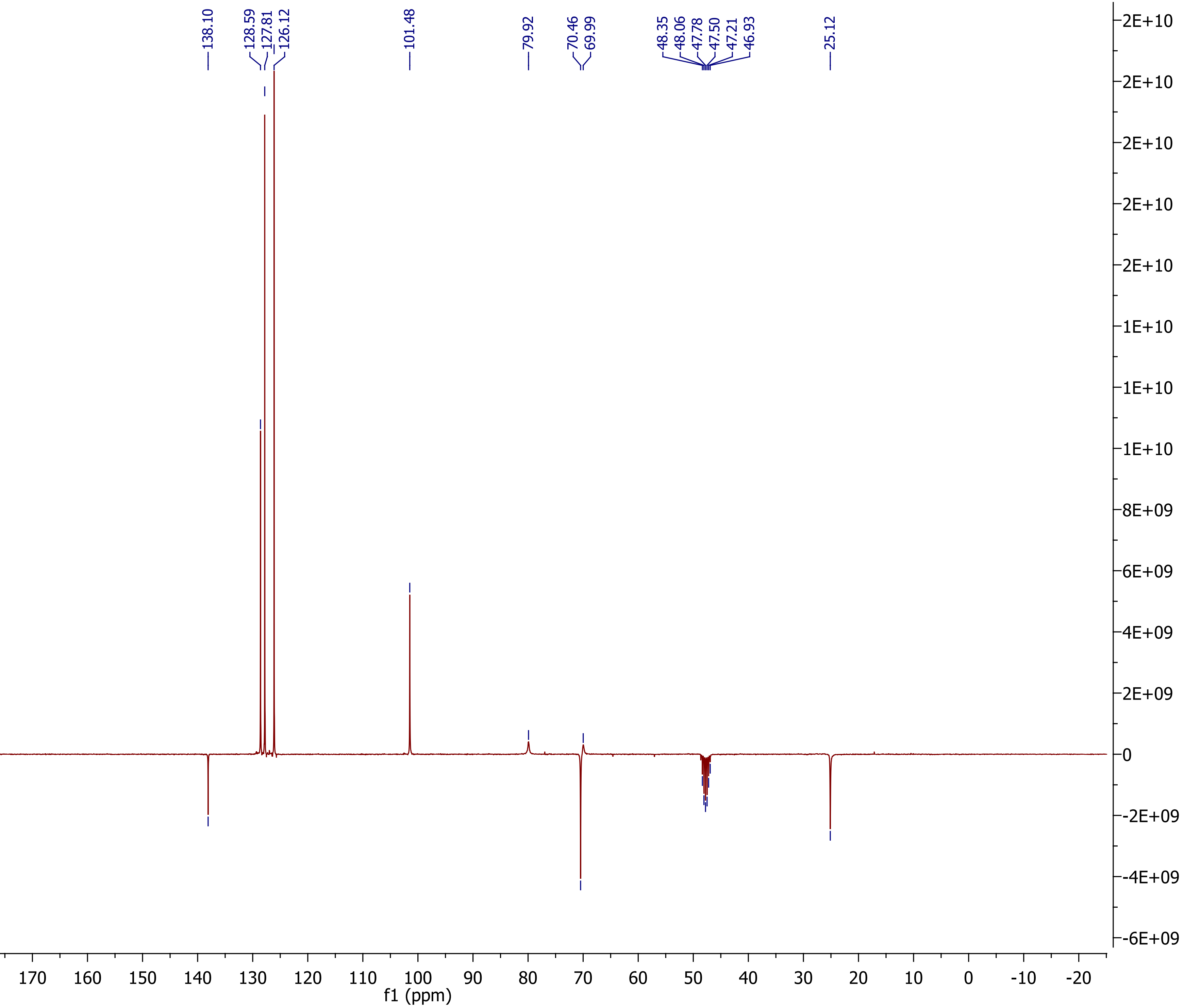

^{11}B NMR (96 MHz, METHANOL-D3)

(10)

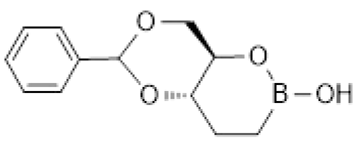

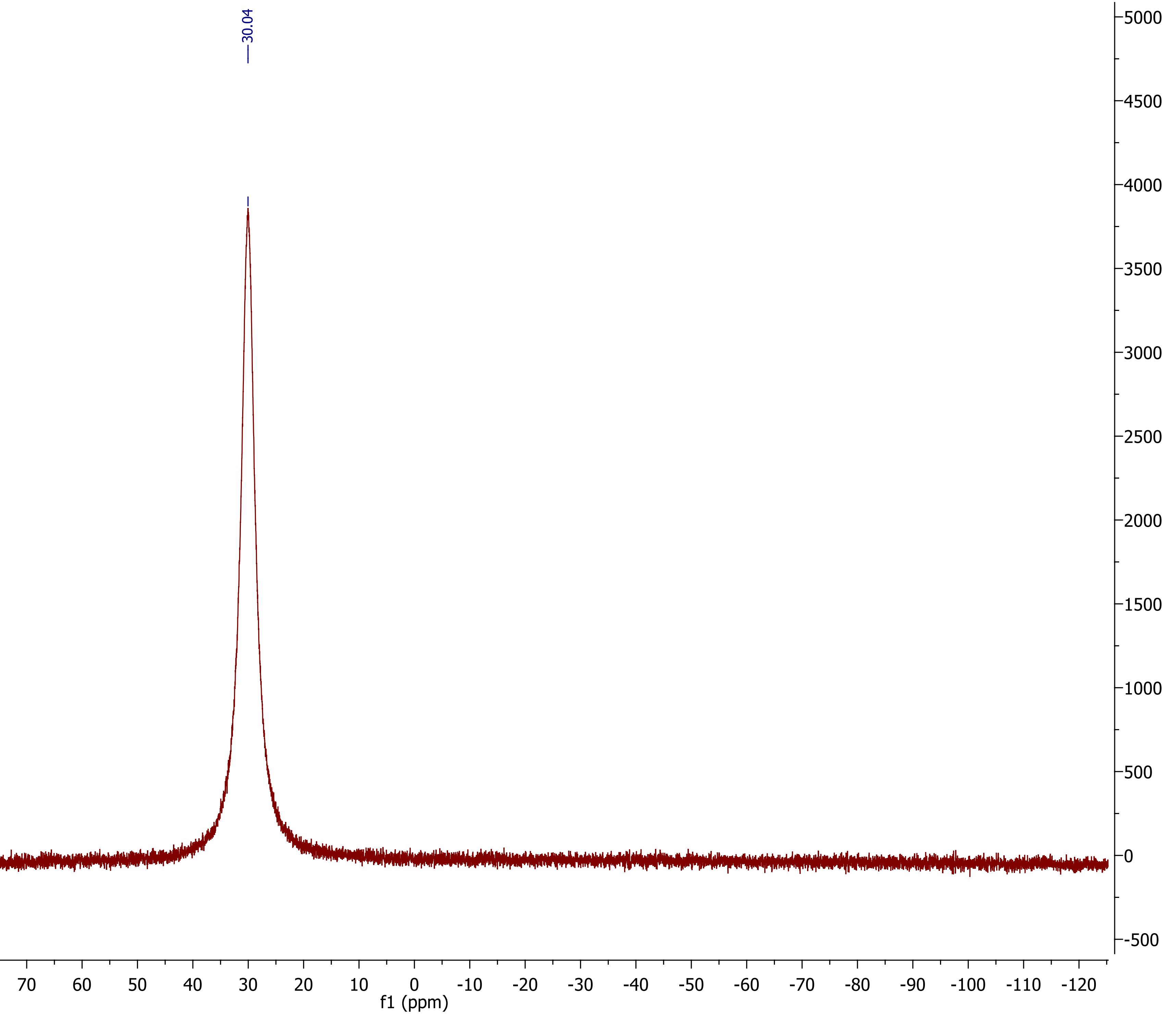

^{1}H NMR (301 MHz, METHANOL-D3)

(11)

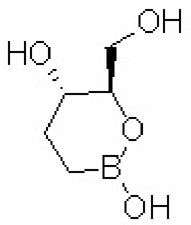

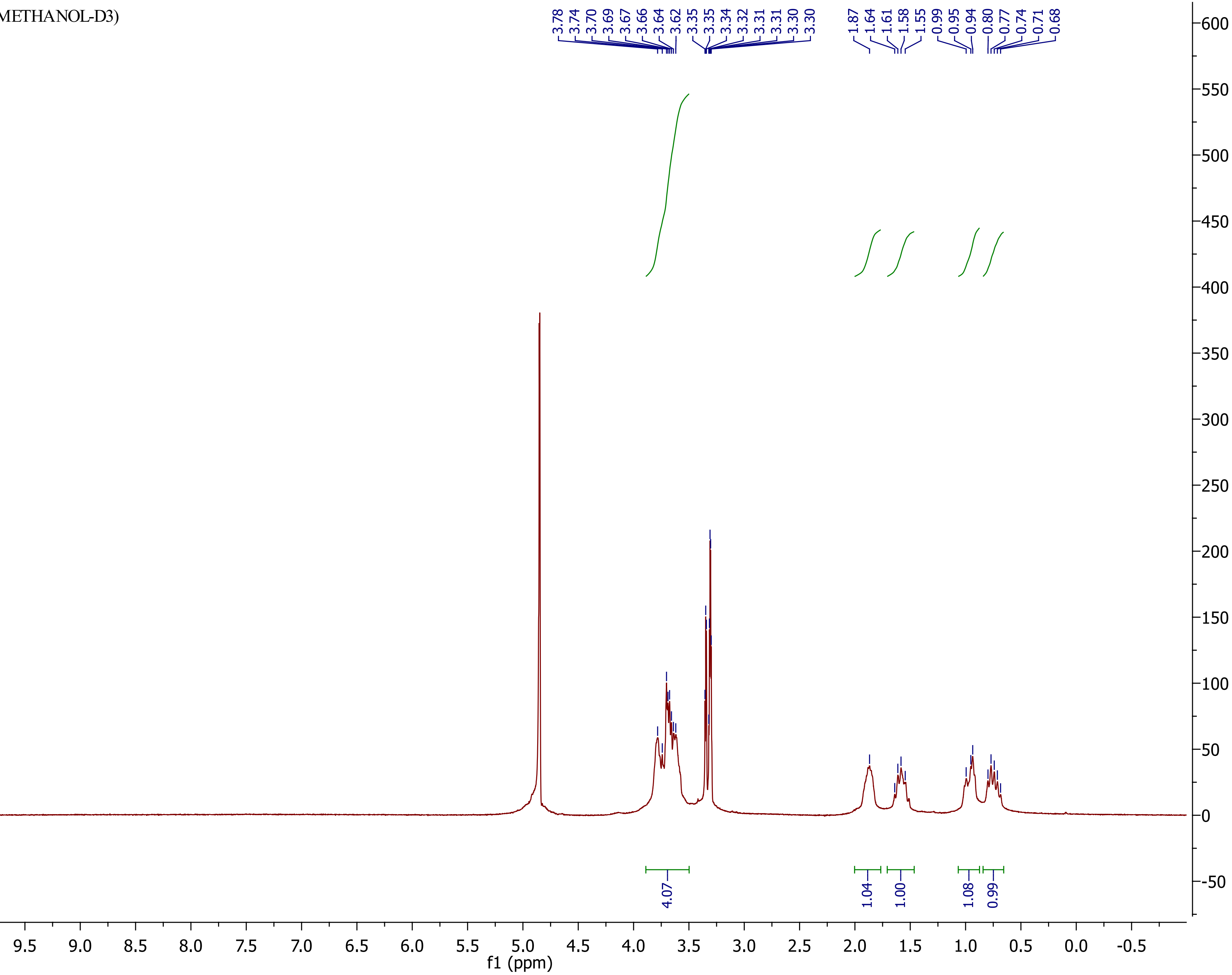

^{13}C NMR (76 MHz, METHANOL-D3)

(11)

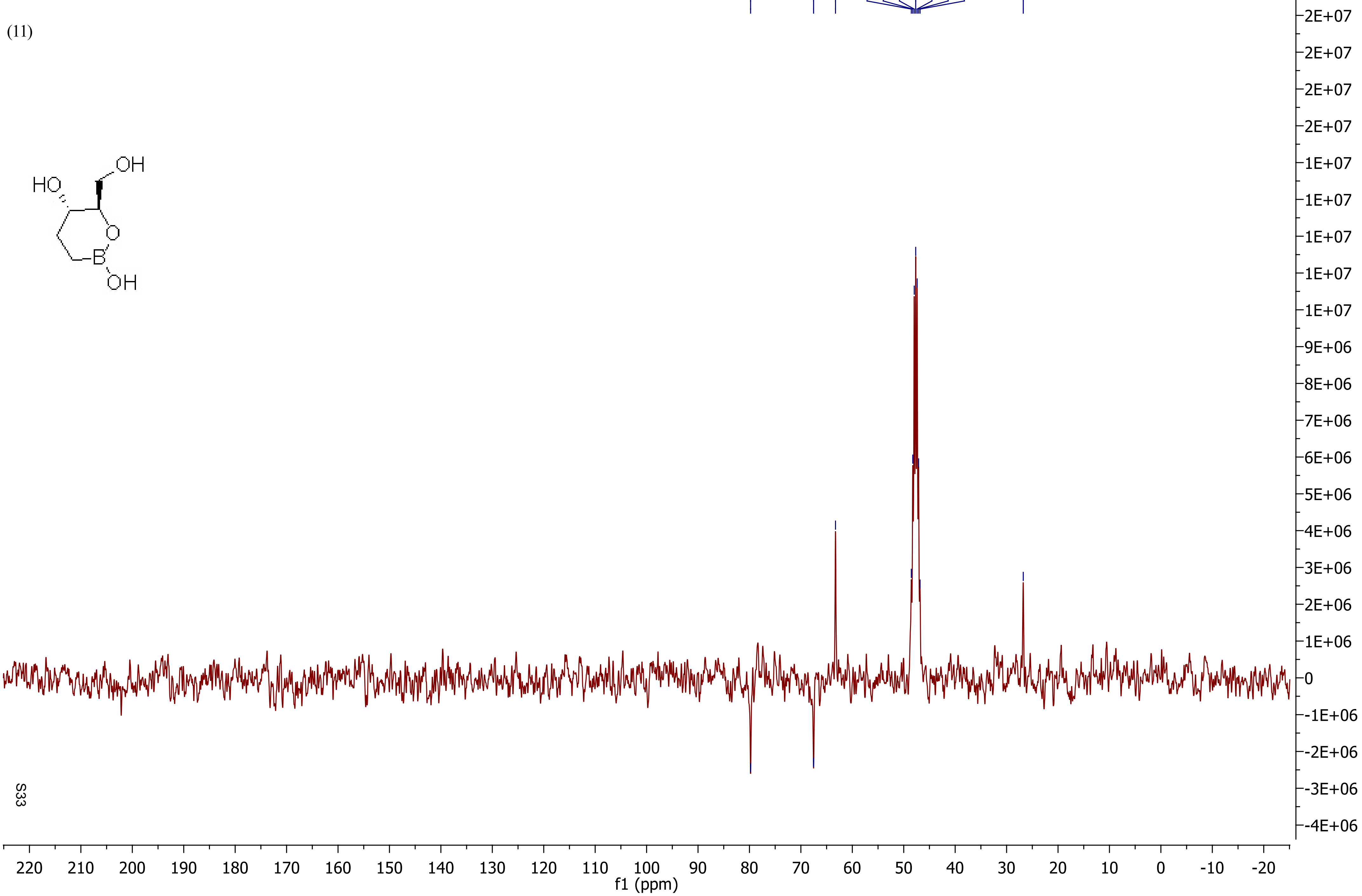

^{11}B NMR (96 MHz, METHANOL-D3)

(11)

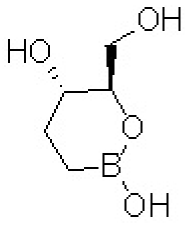

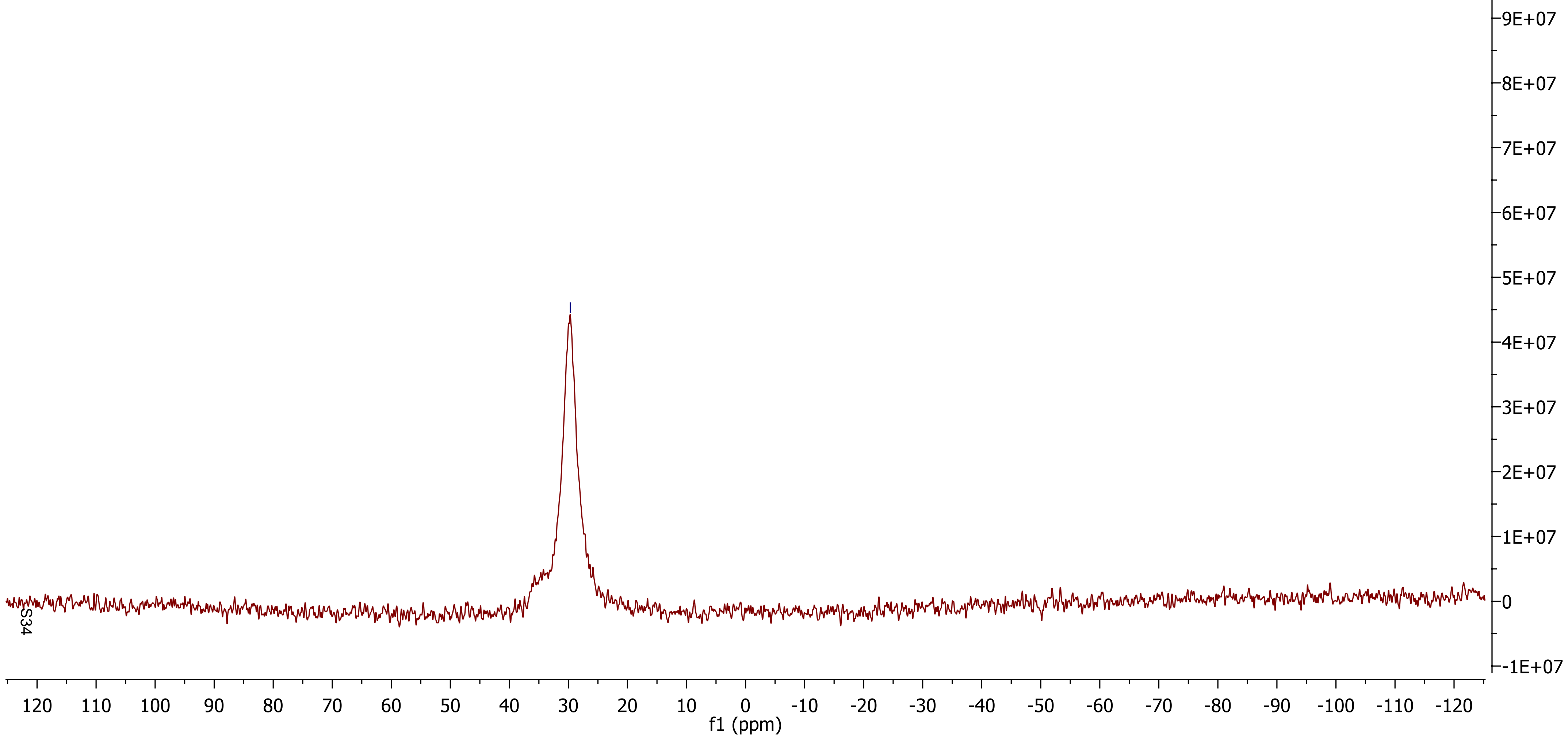

Supplement: File 1 — Experimental procedures and analytical data. Copies of NMR spectra of all new compounds, mass analysis of compounds 8 and 8a and toxicity data. [file Beilstein_J_Org_Chem-15-1355-s001.pdf]
